# Allergic T_H_2 Response Governed by B-Cell Lymphoma 6 Function in Naturally Occurring Memory Phenotype CD4^+^ T Cells

**DOI:** 10.3389/fimmu.2018.00750

**Published:** 2018-04-10

**Authors:** Takashi Ogasawara, Yuko Kohashi, Jun Ikari, Toshibumi Taniguchi, Nobuhide Tsuruoka, Haruko Watanabe-Takano, Lisa Fujimura, Akemi Sakamoto, Masahiko Hatano, Hirokuni Hirata, Yasutsugu Fukushima, Takeshi Fukuda, Kazuhiro Kurasawa, Koichiro Tatsumi, Takeshi Tokuhisa, Masafumi Arima

**Affiliations:** ^1^Department of Respirology (B2), Chiba University Graduate School of Medicine, Chiba, Japan; ^2^Department of Biomedical Science (M14), Chiba University Graduate School of Medicine, Chiba, Japan; ^3^Department of Reproductive Medicine (G4), Chiba University Graduate School of Medicine, Chiba, Japan; ^4^Biomedical Research Center, Chiba University, Chiba, Japan; ^5^Department of Respiratory Medicine and Clinical Immunology, Dokkyo Medical University Koshigaya Hospital, Koshigaya, Japan; ^6^Department of Pulmonary Medicine and Clinical Immunology, Dokkyo Medical University School of Medicine, Mibu, Japan; ^7^Department of Rheumatology, Dokkyo Medical University School of Medicine, Mibu, Japan; ^8^Department of Developmental Genetics, Chiba University Graduate School of Medicine, Chiba, Japan

**Keywords:** B-cell lymphoma 6, naturally occurring memory phenotype T cells, allergy, T_H_2 cells, asthma

## Abstract

Transcriptional repressor B-cell lymphoma 6 (Bcl6) appears to regulate T_H_2 immune responses in allergies, but its precise role is unclear. We previously reported that Bcl6 suppressed IL-4 production in naïve CD4^+^ T cell-derived memory T_H_2 cells. To investigate Bcl6 function in allergic responses in naturally occurring memory phenotype CD4^+^ T (MPT) cells and their derived T_H_2 (MPT_H_2) cells, *Bcl6*-manipulated *mice*, highly conserved intron enhancer (hcIE)-deficient *mice*, and reporter mice for conserved noncoding sequence 2 (CNS2) 3′ distal enhancer region were used to elucidate Bcl6 function in MPT cells. The molecular mechanisms of Bcl6-mediated T_H_2 cytokine gene regulation were elucidated using cellular and molecular approaches. Bcl6 function in MPT cells was determined using adoptive transfer to naïve mice, which were assessed for allergic airway inflammation. Bcl6 suppressed IL-4 production in MPT and MPT_H_2 cells by suppressing CNS2 enhancer activity. Bcl6 downregulated *Il4* expression in MPT_H_2 cells, but not MPT cells, by suppressing hcIE activity. The inhibitory functions of Bcl6 in MPT and MPT_H_2 cells attenuated allergic responses. Bcl6 is a critical regulator of IL-4 production by MPT and MPT_H_2 cells in T_H_2 immune responses related to the pathogenesis of allergies.

## Introduction

Allergic asthma is an inflammatory airway disorder mediated by T_H_2 cells, which produce various effector cytokines (IL-4, IL-5, and IL-13) (1, 2). IL-4 induces signal transducer and activator of transcription (STAT) 6 phosphorylation, causing the protein to translocate to the nucleus, where it induces the expression of *Gata3* (3, 4), a key regulator of T_H_2 cell differentiation. GATA3 facilitates *Il4, Il5*, and *Il13* transcription in T_H_2 cells (3, 4). In mouse and human allergies, IL-4 initiates T_H_2 responses and IgE isotype class switching, whereas IL-5 and IL-13 are important for eosinophil infiltration/activation and increased airway hyperreactivity in allergic asthma (1, 2).

The proto-oncogene product B-cell lymphoma 6 (Bcl6) is a sequence-specific transcriptional repressor (5–9). Tissue hypereosinophilia occurs with increased IL-4, IL-5, and IL-13 production in B-cell lymphoma 6 (*Bcl6*)*-*knockout (KO) mice, suggesting that Bcl6 participates in allergy pathogenesis and that it may be important for reducing T_H_2 immune responses. However, the T cell-intrinsic function of Bcl6 in T_H_2 cell responses remains unclear. Bcl6-binding DNA sequences resemble STAT protein-bound motifs (10), indicating that Bcl6 may repress T_H_2 cytokine expression by competitively inhibiting the binding of STAT factors to GAS sites in target genes (5, 11–13), including T_H_2 cytokine gene loci (14). We previously identified Bcl6/STAT-binding sequences (BSs) (15) in CNS1 (BS1), IL-4 promoter region (BS2), and DNase hypersensitive site 2 (HS2) (BS3, BS4) and HS3 (BS5, BS6) in intron two and the 3′ region of CNS2 (BS7) in the *Il4* locus; BSIL5 sequences in the *Il5* locus (14); and BSIL13 sequences in the *Il13* locus. We, furthermore, reported that Bcl6 repressed *Il4* and *Il5* expression by binding to genomic DNA in naïve CD4^+^ T cell-derived memory (NAM) T_H_2 cells (14, 15), identifying Bcl6 as a critical regulator of T_H_2 cytokine production in memory CD4^+^ T cells in addition to its role in the maintenance and survival of the cells (15–17). Conversely, T follicular helper (T_FH_) cell differentiation may result from Bcl6-mediated suppression of the differentiation of other T_H_ cell lineages *in vivo* (18–20). Thus, the role of Bcl6 in the regulation of T_H_2 cytokine production in pathophysiological settings remains unclear. We focused on a CD4^+^ T cell subset, namely, naturally occurring memory phenotype CD4^+^ T (MPT) cells (21–27). These are derived from CD4^+^ T cells that naturally exhibit memory cell markers (CD44^high^ CD25^−^ CD49b^−^) without antigen stimulation, rather than from memory CD4^+^ T cells differentiated from naïve CD4^+^ T cells after antigen stimulation. A small subset of MPT cells and their derived MPT_H_2 cell populations, but not naïve CD4^+^ T cell-derived T_H_2 cells (NAT_H_2 cells), have an active conserved noncoding sequence 2 (CNS2) 3′ distal enhancer region in the *Il4* locus similar to that in natural killer T cells, producing IL-4 without T cell receptor (TCR)-mediated stimulation (28). CNS2-active MPT cells are candidate cells that initially produce IL-4 to promote T_H_2 cell differentiation, and thus, they may be involved in allergy pathogenesis, although the mechanisms remain unclear. Because Bcl6 expression is extremely high in CNS2-active MPT cells (29), we hypothesized that Bcl6 regulates allergen-mediated MPT cell activation in T_H_2 cell-dependent allergies.

## Materials and Methods

### Antibodies (Abs) and Reagents

Allophycocyanin-conjugated anti-CD4 monoclonal antibody (mAb, GK1.5), anti-IL-4 mAb (11B11), anti-IFN-γ mAb (R4-6A2), anti-CD62L mAb (MEL-14), anti-CD44 mAb (IM7), PE-conjugated anti-IL-4 mAb (BVD4-1D11), PE-conjugated KJ1-26 (anti-clonotypic mAb for DO11.10 TCR, KJ1-26), anti-CD11c mAb (HL3), unconjugated anti-IL-4 mAb (11B11), anti-IL-12 mAb (C17.8), anti-IFN-mAb (R4-6A2), anti-CD44 mAb (IM7), FITC-conjugated anti-CD49b mAb (DX5), and PerCP-conjugated anti-CD4 mAb (GK1.5) were purchased from BD Bioscience. Anti-STAT5 Abs (C-17), anti-STAT6 Abs (N-20), anti-Bcl6 Abs (N-3), anti-tubulin Abs (H-235), and normal rabbit IgG were purchased from Santa Cruz Biotechnology. FITC-conjugated anti-T1/ST2 (IL-33R) mAb (DJ8) was purchased from MD Bioproducts. Mouse rIL-2, rIL-4, rIL-7, rIL-12, and rIL-33 were purchased from PeproTech. Anti-CD3ε mAbs (145-2C11) were purchased from Cedar Lane. Anti-CD28 mAbs (PV-1) were purchased from Southern Biotechnology. The ovalbumin (OVA) peptide (Loh15: residues 323–339; ISQAVHAAHAEINEAGR) was synthesized by BEX Co. Ltd. (Tokyo, Japan). The Bcl6 inhibitory peptide was synthesized by Scrum Inc. (Tokyo, Japan).

### Animals

*Bcl6-*transgenic (TG) mice with exogenous *Bcl6* under Lck proximal promoter control (17, 30), *Bcl6*-KO mice (31), and highly conserved intron enhancer (hcIE)-KO mice on a BALB/c background (Japan SLC) were described previously (15). CNS2-green fluorescent protein (GFP)-TG mice were gifted by Dr. Masato Kubo (28). Some *Bcl6*-TG, *Bcl6*-KO, and hcIE-KO mice were crossed with OVA-specific TCRαβ (DO11.10) and/or CNS2-GFP-TG mice. All mice were used at 8–12 weeks of age.

### CD4^+^ T Cell Purification and T_H_ Cell Induction

Naïve CD44^low^ CD62L^+^ CD4^+^ T cells, CD44^high^ CD62L^−^ CD4^+^ MPT cells, transferred T cells, dendritic cells (DCs), and T cell-deleted splenocytes were isolated from murine spleens using a cell sorter (FACSVantage, BD Biosciences). Sorted T cells (2 × 10^5^ cells/mL) from DO11.10 background mice were stimulated with OVA peptides (Loh15) (1 µg/mL) plus irradiated or CD11C^+^ DCs (4 × 10^4^ cells/mL) or splenocytes (1 × 10^6^ cells/mL), depleted of CD4^+^ and CD8^+^ T cells, and used as antigen-presenting cells (APCs) in the presence of rIL-2 (25 U/mL) (T_H_0 condition). In addition to primary TCR-mediated stimulation with OVA, stimulation with soluble anti-CD3 (2 µg/mL) and anti-CD28 mAbs (2 µg/mL) was employed for some experiments. For T_H_1 or T_H_2 polarization, cells were cultured in the presence of rIL-12 (100 U/mL)/anti-IL-4 mAb (5 µg/mL) or rIL-4 (1,000 U/mL)/anti-IL-12 mAb (10 µg/mL), as previously described (15). In some experiments, anti-IL-4 mAbs or anti-IFN-γ mAbs were added to the T_H_0 condition cultures. On days 3 and 5, activated naïve T cells and MPT cells were stimulated with rIL-2 (25 U/mL) and rIL-7 (10 U/mL) following primary stimulation. NAT_H_2 cells were further cultured with IL-7 for 21 days to yield NAT_H_2 cell-derived memory-like T_H_2 (NAM-LT_H_2) cells, which have a functional phenotype similar to NAT_H_2 cell-derived memory (NAMT_H_2) cells *in vivo* (15). Some MPT cells were cultured in the presence of IL-33 (0–100 ng/mL) with or without IL-7 for the appropriate times as shown in each experiment prior to analysis of chromatin immunoprecipitation (ChIP) assays and the effect of TCR stimulation on cytokine production.

### Fluorescence-Activated Cell Sorting (FACS) Analysis

As previously described (15, 17), T cells with or without 8 h of restimulation were treated with monensin (2 µM) for the last 3 h, followed by staining with an appropriate combination of FITC-conjugated anti-KJ1-26, APC-conjugated anti-CD44, and PerCP-conjugated anti-CD4 mAbs. For staining, cells were washed once with FACS buffer (PBS with 3% fetal calf serum and 0.1% sodium azide) and then permeabilized with Perm2 (BD Biosciences) for 10 min at room temperature, followed by two washes in FACS buffer. Finally, cells were stained with an appropriate combination of anti-IFN-γ-APC and anti-IL-4-PE for 30 min at room temperature, washed, and resuspended in FACS buffer for analysis.

### Cytokine Concentrations

IL-4, IL-5, and IL-13 levels in the culture supernatants of cells that were stimulated for 48 h in bronchoalveolar lavage fluid (BALF) were determined using ELISA kits (R&D Systems, Minneapolis, MN, USA). IgE anti-OVA Abs were detected using a mouse anti-OVA IgE Antibody Assay Kit (Chondrex, Redmond, WA, USA).

### mRNA Measurements

cDNA synthesized from total RNA using the SuperScript III First-Strand Synthesis System (Invitrogen) was used for qRT-polymerase chain reaction (PCR) analysis as described previously (15). Real-time PCR was performed in 25 µL reaction volumes containing iQ SYBR-Green Supermix, 200 nM of each primer, and 0.5 µL of cDNA. The PCR cycle parameters were 3 min at 95°C and 40 cycles of 30 s at 95°C, 30 s at 60°C, and 30 s at 72°C, followed by melting curve analysis. Relative quantification of cytokine mRNA expression was performed using the comparative Ct method. The relative quantification value of the target in stimulated T cells, normalized to the β-actin gene expression level (endogenous control) and relative to a calibrator, was expressed as 2^−ΔΔCt^ (fold), where ΔCt = Ct of the target gene − Ct of the endogenous control gene (β-actin) and ΔΔCt = ΔCt of stimulated samples for target gene − ΔCt of the untreated control as a calibrator for the target gene. All data in stimulated T cells were expressed as arbitrary units relative to the expression level in the corresponding unstimulated T cells. The primers were as follows: β*-actin*: 5′-CCAGCCTTCCTTCTTGGGTAT-3′ (forward), 5′-TGGCATAGAGGTCTTTACGGATGT-3′ (reverse); *Il4*: 5′-TCTCGAATGTACCAGGAGCCATATC-3′ (forward), 5′-AGCACCTTGGAAGCCCTACAGA-3′ (reverse); *Il5*: 5′-CGATGAGGCTTCCTGTCCCTA-3′ (forward), 5′-TTGGAATAGCATTTCCACAGTACCC-3′ (reverse); *Il13*: 5′-CAATTGCAATGCCATCTACAGGAC-3′ (forward), 5′-CGAAACAGTTGCTTTGTGTAGCTGA-3′ (reverse); *Gata3*: 5′-AGAGATTTCAGATCTGGGCAATGG-3′ (forward), 5′-CAGGGACTGATTCACAGAGCATGTA-3′ (reverse); *Bcl6*: 5′-CCGGCTCAATAATCTCGTGAA-3′ (forward), 5′-GGTGCATGTAGAGTGGTGAGTGA-3′ (reverse).

### Chromatin Immunoprecipitation

The ChIP assay was performed as previously described (14, 15). Protein and chromatin in T_H_ cells were cross-linked by adding formaldehyde solution (Thermo Fisher Scientific, Waltham, MA, USA), after which the cells were lysed in SDS lysis buffer. Subsequently, precleared, sonicated chromatin and protein G agarose (Millipore) were incubated with specific Abs for the protein of interest or control IgG (rabbit). Some of the untreated chromatin was used as an input sample. qPCR was used to quantify the DNA region in the immune-precipitated chromatin and the input DNA. Relative ChIP DNA quantification was performed using the comparative Ct method. The Ct value of ChIP DNA was normalized to that of the input DNA using the following equation: ΔCt (normalized ChIP) = Ct (ChIP) − Ct (input). The normalized Ct values were adjusted to the normalized background Ct value (ΔΔCt [ChIP/IgG] = ΔCt [normalized ChIP] − ΔCt [normalized IgG]). ChIP enrichment above the sample specific background was calculated as 2^−ΔΔCt (ChIP/IgG)^ and reported as a fold change. The following primers were used for qPCR: *Il5BS*: 5′-TGGGCCTTACTTCTCCGTGTAACT-3′ (forward), 5′-CTCCAGTGACCCTGATACCTGAAT-3′ (reverse); *Il13BS*: 5′-TTCTACTAGCTCGGGACTCTTCCA-3′ (forward), 5′-ATGGACATGACATGGGAAACCCAG-3′ (reverse); *BS1*: 5′-AGGTCCATGGAAGGGACAGATCA-3′ (forward), 5′-CGGATCCTTTCCTGGAATTGCTGA-3′ (reverse); *BS2*: 5′-TCCAATTGGTCTGATTTCACAGGA-3′ (forward), 5′-ACACCAGATTGTCAGTTATTCTGGGC-3′ (reverse); *BS3*: 5′-ACAGATGTGACAGGCTGATAGTGC-3′ (forward), 5′-GGCCTTTCATTCTCAGTGGTGTGT-3′ (reverse); *BS4*: 5′-CCTGGCTTCTGAGATGCAATGAGT-3′ (forward), 5′-GGGTAAGAGGAAAGCCAGCATGA-3′ (reverse); *BS5*: 5′-TTCAAGGATAAGCAAGTGGCAGGC-3′ (forward), 5′-ATTGGAACTAAGCCAGCCGATGGA-3′ (reverse); *BS6*: 5′-CGCCTCTCCTGTAAGGTACACAAT-3′ (forward), 5′-TTGCCTTGCAACCATGAAGACCTG-3′ (reverse); *BS7*: 5′-CACTCACCAATTTGTCTGGAGGCT-3′ (forward) 5′-ATGGTGATCACAGTCCAAGTCCAG-3′ (reverse).

### Retroviral Vectors With a *d2EGFP* Reporter Gene

A genomic fragment of the *Il4* promoter (p) region (positions −751 to +1 relative to the transcription start site, MGI: 96556), hcIE (222 bp), and CNS2 (337 bp) were amplified by PCR. The fragment of the *Il4* promoter region was subcloned upstream of *d2-enhanced GFP* (*d2EGFP*) in the retrovirus vector pBABE delta Bll(−). Fragments of hcIE (222 bp) or CNS2 (337 bp) were subcloned downstream of *d2EGFP* to generate pBABE delta Bll(−)-*Il4*p-*d2EGFP*-hcIE or pBABE delta Bll(−)-*Il4*p-*d2EGFP*-CNS2, respectively. pBABE delta BII(−) is based on pBABEpuro (3) (gifted by Dr. H. von Melchner, University of Frankfurt Medical School). PCR-based mutagenesis of G3 and BS3 in hcIE and BS7 (1) and (2) in CNS2 was achieved using a QuickChange XL Site-Directed Mutagenesis Kit (Stratagene). Specifically, a fragment of *d2EGFP* cDNA was PCR amplified using an *Xho*I-anchored sense primer (underlined) (5′-CCGCTCGAGTCTAGAGGATCCACCGGTCGC-3′) immediately upstream of the *Xba*I site (+258) and an antisense primer with a *Sal*I-anchored antisense primer (underlined) (5′-ACGCGTCGACTCTAGAGTCGCGGCCGCATC-3′) immediately downstream of the *Xba*I site (+1147) of pd2EGFP. The *Xho*I/*Sal*I fragment of *d2EGFP* was subcloned into a T Easy vector (d2EGFP-T vector). The *Eco*RI-digested *d2EGFP* fragment was blunted and subcloned into a blunted *Not*I/*Nco*I-restricted pMX vector (pMX-d2EGFP). A genomic fragment of the *Il4* promoter region was PCR amplified using the *Eco*RI-anchored sense primer (underlined) (5′-GAATTCCTCCACACTGATGCTGTAGTGC-3′) and *Xho*I-anchored antisense primer (underlined) (5′-CTCGAGGCTAACAATGCTGGC-3′). The subcloned *Il4* promoter fragment was then digested with *Eco*RI and *Xho*I and subcloned into the restricted site of pMX-d2EGFP (pMX-*Il4*p-*d2EGFP*). An *Eco*RI and *Sal*I fragment of pMX-*Il4*p-*d2EGFP* was then subcloned into the *EcoR*I/*Sal*I-restricted pBABE delta Bll(−) to generate pBABE delta Bll(−)-*Il4*p-*d2EGFP*. The vector pBABE delta BII(−) is based on pBABEpuro, with further modifications to completely destroy the endogenous transcriptional regulatory sequences within the retroviral long terminal repeat (LTR). R and U5 are the intact R and U5 regions of MMLV, respectively, en. del. U3 is the SIN U3 found in proviral LTRs after integration of the virus into the host genome, and partial LTR denotes a transcription-competent part of the LTR that is used to drive transcription of the genomic viral RNA in the packaging cells. The hcIE genomic fragments were PCR amplified with the *Xho*I-anchored sense primer (underlined) (5′-CCGCTCGAGCCTTTCTGCCTGCTGCTCTG-3′) and *Sal*I-anchored antisense primer (underlined) (5′-ACGCGTCGACGAAAAGCAGGCAGTCTGGAG-3′).

Conserved noncoding sequence 2 fragments were obtained by PCR using the *Xho*I-anchored sense primer (underlined) (5′-CCGCTCGAGCTGGAGATTAGAAGTGGAGGCT-3′) and *Sal*I-anchored antisense primer (underlined) (5′-ACGCGTCGACTTTCCTGTCCTCGTCTTTTCCAGT-3′). The hcIE and CNS2 fragments were then inserted in *Sal*I-digested pBABE delta Bll(−)-*Il4*p-*d2EGFP* to generate pBABE delta Bll(−)-*Il4*p-*d2EGFP-*hcIE and pBABE delta Bll(−)-*Il4*p-*d2EGFP-*CNS2, respectively, for reporter gene assays. PCR-based mutagenesis of G3 (5'-CTGATAGTG-3′: +1247 to +1255), BS3 (5′-TTCATGGAA-3′: +1328 to +1336) in hcIE, and BS7 (1) (5′-GTTTTTGAA-3′: +12941 to +12949) and BS7 (2) (5′-TTCCTGGA-3′: +13142 to +13149) in CNS2 in the reporter plasmid were generated using a QuickChange XL Site-Directed Mutagenesis Kit according to the manufacturer’s instructions. The underlined nucleotides were substitutes for CTAT for G3 and TT for BS3 and BS7 to generate pBABE delta Bll(−)-*Il4*p-*d2EGFP-*hcIE-MutBS3, pBABE delta Bll(−)-*Il4*p-*d2EGFP-*hcIE-MutG3, pBABE delta Bll(−)-*Il4*p-*d2EGFP-*CNS2-MutB7 (1), and pBABE delta Bll(−)-*Il4p-d2EGFP-*CNS2-MutB7 (2), respectively. Successful PCR and mutation were verified by DNA sequencing.

### Retrovirus Infection

Platinum-E packaging cells (32) were transfected with 1–1.5 µg of DNA of a retrovirus construct mixed with 6 µL of Fugene (Boehringer Mannheim). Virus supernatant was concentrated by centrifugation (8,000 × *g*, 16 h) and added to T_H_2 cell-inducing cultures on day 2. Intracellular cytokine staining or mean fluorescence intensity (MFI) analysis was performed on day 7 as described previously. Infected cells were subjected to FACS analysis of the intracellular fluorescence of d2EGFP 8 h after restimulation with plate-bound anti-CD3 mAbs.

### Western Blot Analysis

*In vitro*-differentiated T_H_2 cells were lysed with lysis buffer (1% Nonidet P-40, 5% glycerol, 50 mM Tris–HCl, pH 7.5, 100 mM NaCl, 10 µg/mL leupeptin, 0.1 mM phenylmethylsulfonyl fluoride, 1 mM dithiothreitol, 1 µg/mL pepstatin A, 10 mM Na_3_VO_4_, and 10 mM NaF). For immunoblotting, anti-Bcl6 or anti-β-tubulin Ab was used. Immunoreactive bands were visualized using a Phototope-HRP Western Blot Detection System (Cell Signaling Technology). For quantitative analysis of Western blots, the intensities of individual bands were quantified using ImageJ software (National Institutes of Health, Bethesda, MD, USA).

### Antigen-Induced Airway Inflammation

#### OVA Challenge and Bronchoalveolar lavage (BAL)

T_H_2 cells (1.5 × 10^7^ or 3 × 10^7^) were injected intravenously into naïve wild-type (WT) BALB/c mice (day 0), followed by intratracheal challenge with 1% OVA solution (50 µL) twice (days 2 and 3), BAL three times (days 2, 7, and 12), and serum collection. On days 4 and 5, the transferred T_H_2 cells isolated from whole lungs and BALF were collected from the mice by instilling the lungs with 0.5 mL of PBS six times. Sera on day 14 were analyzed for OVA antigen-specific IgE Abs. In another experiment, a mixture of *Bcl6-*WT, *Bcl6-*TG, or *Bcl6-*KO KJ1-26^+^ MPT (2 × 10^6^ cells) and *Bcl6-*WT KJ1-26^−^ naïve CD4^+^ T (5 × 10^6^ cells) cells were intravenously transferred into BALB/c *nu*/*nu* mice (day 0). Subsequently, mice were sensitized *via* i.p. injection of 10 µg of OVA plus 1 mg of alum twice (days 1 and 6), followed by intratracheal challenge with OVA twice (days 16 and 17). BAL and pathology examination were performed (day 18), and transferred KJ1-26^−^ cells were isolated from spleens (day 16). The isolated cells were restimulated with plate-bound anti-CD3 mAbs to analyze cytokine production. The BALF supernatant was stored at −80°C. Each cell pellet was resuspended in PBS for counting and subjected to cytospin. Preparations on slides were stained with Diff-Quick (Sysmex International Reagents, Kobe, Japan) for the differential analysis of cell counts. After BAL, lungs were treated with collagenase II (1 mg/mL) for 30 min at 37°C, and leukocytes were isolated on a Percoll gradient.

#### Histologic Examination

After BAL, the left lobes of lungs were extracted, washed with PBS, and fixed in 4% formaldehyde in sodium phosphate buffer for more than 2 days at room temperature. After fixation, lungs were embedded in paraffin and stained with hematoxylin and eosin. Images of each tissue section were captured using a Zeiss Axioscope 2 microscope equipped with a video camera (AxioCam ERc5s, Carl Zeiss, Jena, Germany) and processed using Axiovision V.4 software (Carl Zeiss).

### Statistical Analysis

Statistical significance was determined using *t*-tests (two-tailed) for two groups and Tukey–Kramer or Steel–Dwass multiple comparisons tests for three or more groups. *P* values < 0.05 were considered significant.

## Results

### Bcl6 Represses IL-4 Production by MPT Cells

**Figure 1:**
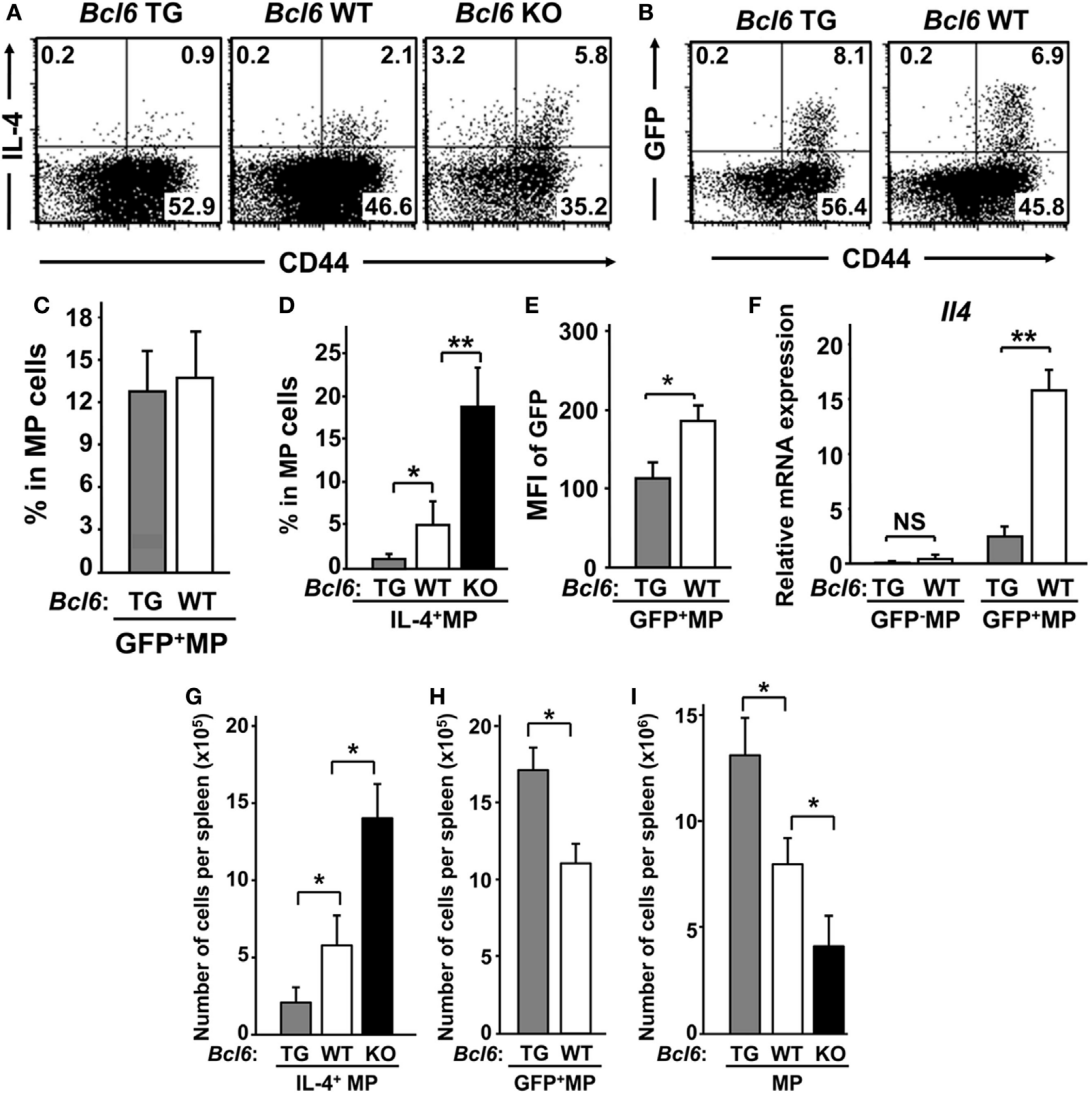
IL-4^+^ MPT cells in mice with varying genetic *Bcl6* expression. **(A,B)** FACS analysis of intracellular IL-4^+^ [**(A)** *Bcl6*-TG, *Bcl6*-WT, and *Bcl6*-KO] and CNS2-activation-related GFP^+^ [**(B)** *Bcl6*-TG and *Bcl6*-WT] MPT cells in a CD44^high^ population by gating CD4^+^ CD49b^−^ T splenocytes at rest. The presented data are representative of four independent experiments. The numbers in the corners represent the percentages of gated T cells. **(C,D)** Frequency of GFP^+^ [**(C)** *Bcl6*-TG and *Bcl6*-WT] and IL-4^+^ [**(D)** *Bcl6*-TG, *Bcl6*-WT, and *Bcl6*-KO] MPT cells. **(E)** MFI of GFP in MPT cells from *Bcl6*-TG and *Bcl6*-WT mice. **(F)** qRT-PCR analysis of the relative expression of *Il4* in GFP^−^ and GFP^+^ MPT cells from *Bcl6*-TG and *Bcl6*-WT spleens. **(G–I)** Absolute cell numbers of populations of IL-4^+^ [**(G)** *Bcl6*-TG, *Bcl6*-WT, and *Bcl6*-KO], GFP^+^ [**(H)** *Bcl6*-TG and *Bcl6*-WT], and total **(I)** MPT cells in one spleen. Data are presented as the mean ± SEM (*n* = 7–9). *P < 0.05; **P < 0.01, comparison between two groups as indicated. Bcl6, B-cell lymphoma 6; CNS, conserved noncoding sequence; FACS, fluorescence-activated cell sorting; GFP, green fluorescent protein; KO, knockout; MFI, mean fluorescence intensity; MPT cell, memory phenotype CD4^+^ T cell; NS, not significant; TG, transgenic; WT, wild-type.

Splenic CNS2-active MPT cells were detected as a GFP^+^ subpopulation in reporter gene TG mice (CNS2-GFP-TG) on each *Bcl6* genotype background (28) (Figure 1A). Unfortunately, offspring from CNS2-GFP-TG mice on the *Bcl6*-KO background could not be obtained (Figure 1B). Although the percentages of GFP^+^ cells were similar between *Bcl6*-TG and *Bcl6*-WT mice (Figure 1C), the IL-4^+^ MPT cell frequency (Figure 1D) and MFI of CNS2-GFP in MPT cells (Figure 1E) were inversely correlated with Bcl6 levels. GFP^+^ MPT cells displayed significant *Il4* expression, which was lower in *Bcl6*-TG cells than in WT cells (Figure 1F). *Il4* expression was extremely low in the GFP^−^ population regardless of Bcl6 levels. The absolute numbers and percentages of IL-4^+^ MPT cells were also negatively associated with Bcl6 levels (Figure 1G), whereas the absolute numbers of GFP^+^ MPT cells (Figure 1H) and MPT cells (Figure 1I) among all CD4^+^ T cells were positively correlated with Bcl6 levels. Therefore, Bcl6 may be involved in *Il4* downregulation in MPT cells and MPT cell survival and maintenance. Because it has been reported that the T_H_2 and T_H_1 conditions are promotive and inhibitory, respectively, on the maintenance of *Bcl6-*WT CNS2-GFP^+^ MPT cells (28), we analyzed the effect of Bcl6 on the maintenance of CNS2-GFP^+^ MPT cells in each culture setting (Figure S1 in Supplementary Material). Regarding the maintenance of GFP^+^ cells, a promoting effect of the T_H_2 condition and inhibitory effect of T_H_1 condition were observed regardless of the *Bcl6* genotype, whereas Bcl6 appears to function as a suppressor for CNS2 activity.

### Bcl6 Represses *Il4* Expression in T_H_2-Primed MPT Cells

**Figure 2:**
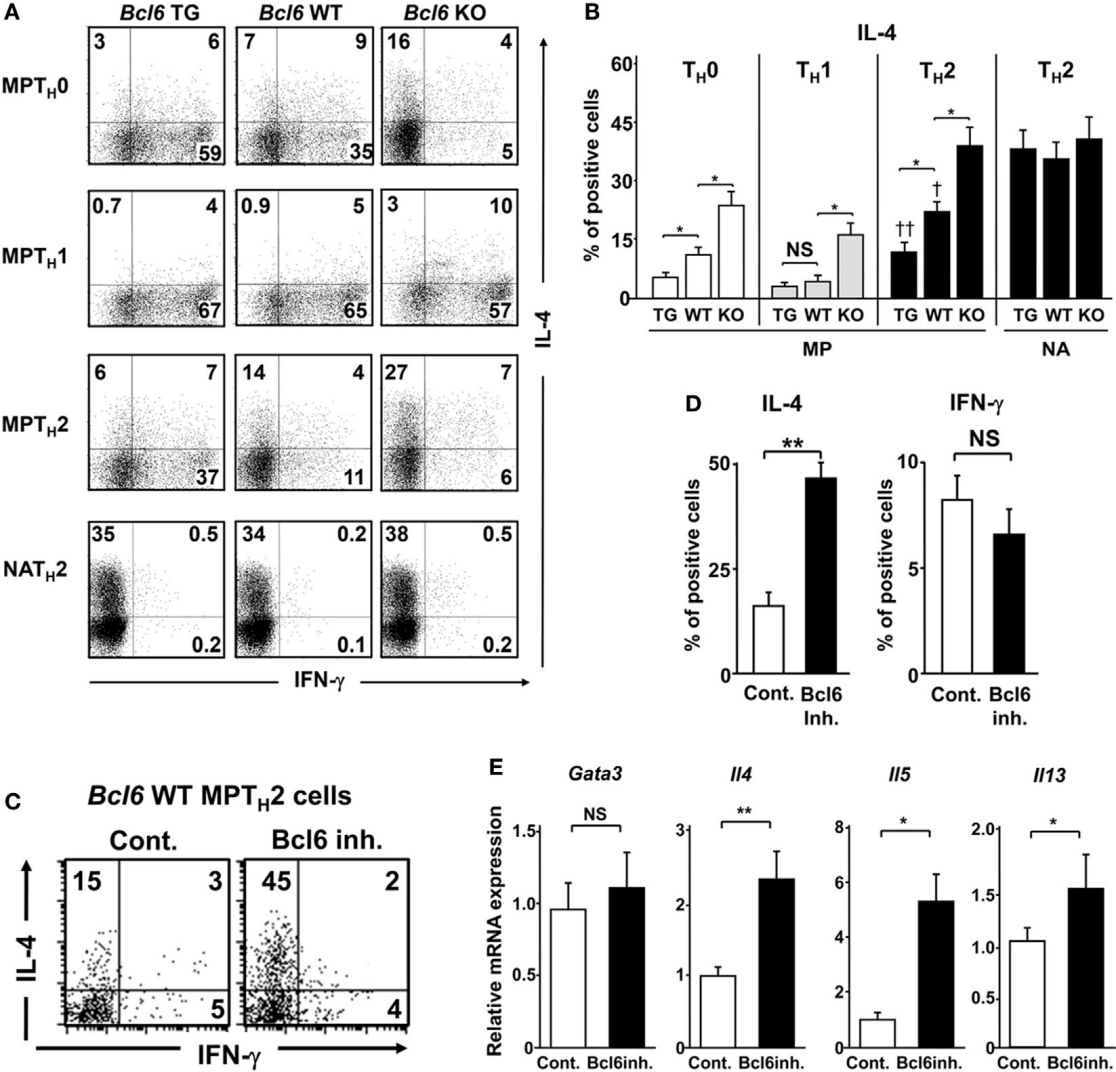
Regulatory role of Bcl6 in the differentiation of T_H_2 cells. **(A*–*E)** KJ1-26^+^ MPT and NA T cells (CD4^+^ CD44^l^°^w^ CD62L^+^) among splenocytes from *Bcl6*-TG, *Bcl6*-WT, and *Bcl6*-KO DO11.10 mice cultured with ovalbumin peptides and antigen-presenting cells *in vitro* for 7 days to produce T_H_0, T_H_1, and T_H_2 cells. Cells were restimulated with anti-CD3 monoclonal antibodies. After 8 h, IL-4- and IFN-γ-producing cells among gated KJ1-26^+^ CD4^+^ T cells were analyzed by FACS. **(C*–*E)** Analysis of cytokine production by *Bcl6*-WT MPT_H_2 cells treated with a Bcl6 inhibitor (inh.) for 12 h prior to restimulation. **(A,C)** Numbers in the corners represent percentages among gated T cells. **(B,D)** Percentage of IL-4^+^ (*Bcl6*-TG, *Bcl6*-WT, and *Bcl6*-KO) cells for each T_H_ cell type **(B)** and IL-4^+^ and IFN-γ^+^ *Bcl6*-WT MPT_H_2 cells cultured with or without Bcl6 inhibitor **(D)**. **(E)** qRT-PCR analysis of the relative expression of *Gata3, Il4, Il5*, and *Il13* in restimulated *Bcl6*-WT MPT_H_2 cells treated with or without a Bcl6 inhibitor. Data are presented as the mean ± SEM (*n* = 7–8). *P < 0.05, **P < 0.01, comparison between two groups is indicated; **^†^***P* < 0.05, compared with *Bcl6*-WT. All results are representative of five independent experiments with similar outcomes, excluding **(C)**, for which four experiments were conducted. Bcl6, B-cell lymphoma 6; Cont., control; FACS, fluorescence-activated cell sorting; KO, knockout; MPT cell, memory phenotype CD4^+^ T cell; MPT_H_2 cell, MPT cell-derived T_H_2 cell; NA, naïve; TG, transgenic; WT, wild-type.

To investigate the function of Bcl6 in the differentiation of MPT cells into T_H_ cell lineages following TCR stimulation, MPT cells expressing a clonotypic TCR (KJ1-26^+^) from the spleens of *Bcl6*-TG, *Bcl6*-KO, and *Bcl6*-WT DO11.10 TG mice were cultured under conditions driving them toward the T_H_0, T_H_1, or T_H_2 phenotype, followed by intracellular IL-4 analysis after restimulation with anti-CD3 mAbs (Figures 2A,B). Under the T_H_0 condition, Bcl6 decreased IL-4 production in a concentration-dependent manner, and high Bcl6 expression facilitated IFN-γ induction during T_H_1 phenotype differentiation. Under the T_H_1 condition, Bcl6 deficiency in MPT cells preserved IL-4 production, although its level was lower than that under the T_H_0 condition. Under the T_H_2 condition, Bcl6 negatively regulated MPT cell-derived T_H_2 (MPT_H_2) cell differentiation but not NAT_H_2 differentiation, as previously reported (15) (Figures 2A,B), although Bcl6 could suppress the initial IL-4 production by naïve CD4 T cells under the T_H_0 condition even when blocking the effects of IFN-γ (Figure S2 in Supplementary Material). Because Bcl6 appears to promote IFN-γ production, which may indirectly affect IL-4 induction, we analyzed a mixed culture of *Bcl6*-WT MPT cells with either *Bcl6-*TG or *Bcl6*-KO cells under the T_H_0 condition. *Bcl6*-KO MPT cells caused WT cells to skew clearly toward the T_H_2 phenotype with reduced T_H_1 skewing, whereas *Bcl6*-TG cells promoted slight T_H_ skewing (Figure S3 in Supplementary Material), indicating that increased IL-4 production in *Bck6*-KO MPT cells autoaccelerates T_H_2 cell differentiation by preventing T_H_1 cell differentiation. Thus, Bcl6 appears to promote IFN-γ production by inhibiting IL-4 production rather than inhibiting IL-4 production *via* the promotion of IFN-γ production.

To confirm the suppressive effects of Bcl6 on T_H_2 cytokine genes in MPT_H_2 cells, *Bcl6*-WT MPT_H_2 cells were treated with a Bcl6 inhibitor (15), followed by restimulation with anti-CD3 mAbs. Bcl6 inhibition augmented IL-4 production but not IFN-γ production (Figures 2C,D). T_H_2 cytokine gene expression was upregulated by the inhibitor without changes in *Gata3* expression (Figure 2E), indicating that Bcl6 suppresses *Il4* expression in developing and differentiated MPT_H_2 cells.

### Bcl6 Negatively Regulates the Histone Modification of T_H_2 Cytokine Loci in MPT_H_2 Cells

**Figure 3:**
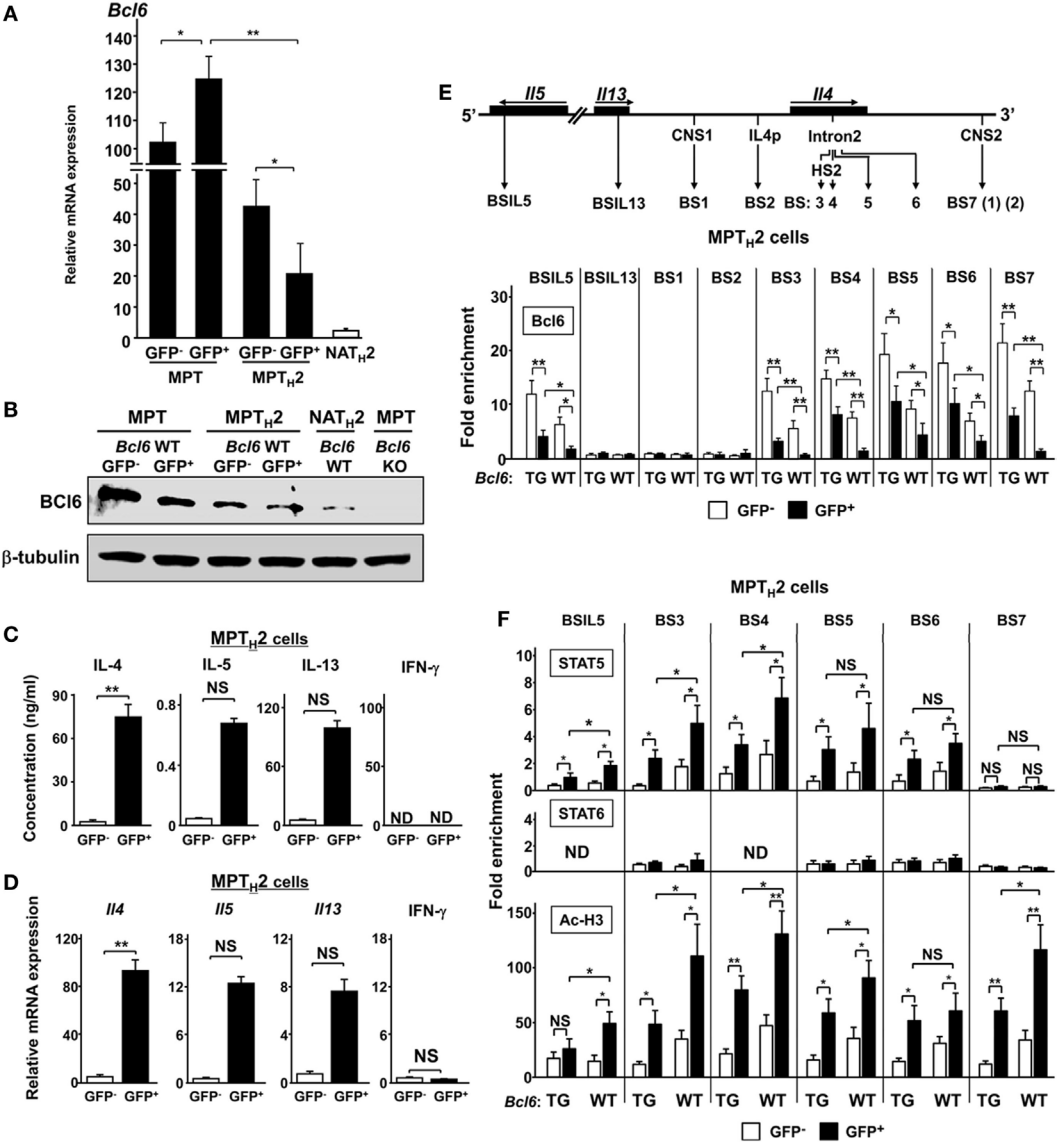
Role of Bcl6 and signal transducer and activator of transcription (STAT) binding to the *Il4* locus MPT cells. **(A)** *Bcl6* mRNA levels in GFP^+^ and GFP^−^ MPT cells, GFP^+^ and GFP^−^ MPT_H_2 cells, and NA T_H_2 cells, as measured by qRT-PCR. **(B)** Western blot analysis of Bcl6 protein in GFP^+^ and GFP^−^ MPT cells MPT (*Bcl6*-WT) and MPT cells (*Bcl6*-KO) in the spleen and GFP^+^ and GFP^−^ MPT_H_2 cells (*Bcl6*-WT). Data are representative of three independent experiments. **(C,D)** KJ1-26^+^ cells among MPT cells from the spleens of *Bcl6*-WT-CNS2-GFP-TG DO11.10 mice were cultured with ovalbumin peptides and antigen-presenting cells *in vitro* for 7 days under T_H_2 conditions. Cells were restimulated with anti-CD3 and anti-CD28 monoclonal antibodies. After 48 h, IL-4, IL-5, IL-13, and IFN-γ levels in culture supernatants were measured by ELISA **(C)**. After 8 h, the mRNA levels of *Il4, Il5, Il13*, and *Ifn-*γ were measured by qRT-PCR **(D)**. [**(E)**, top] Diagram of T_H_2 cytokine gene loci, with regulatory regions indicated by arrows [CNS, gene promoter regions (p), and Bcl6/STAT (BSs): IL5BS in *Il5*; IL13BS in *Il13* intron 1; BS1 and BS7 (1) (2) in CNS1 and CNS2, respectively; BS2 in *Il4*p; and BS3, BS4, and BS5 in *Il4* intron 2]. **(E,F)** Bcl6 levels [**(E)** bottom], STAT5 and STAT6 binding, and Ac-H3 **(F)** at each BS were analyzed by chromatin immunoprecipitation assay for CNS2-active (GFP^+^) (closed bar) and CNS2-inactive (GFP^−^) (open bar) MPT_H_2 cells. All results are representative of three **(A,C,D)** or four **(E,F)** independent experiments with similar outcomes. Data are presented as the mean ± SEM (*n* = 7–9). *P < 0.05, **P < 0.01, comparison between two groups is indicated. Ac-H3, acetylated histone H3; CNS, conserved noncoding sequence; BS, binding sequence; Bcl6, B-cell lymphoma 6; GFP, green fluorescent protein; KO, knockout; ND, not detected; MPT cell, memory phenotype CD4^+^ T cell; MPT_H_2 cell, MPT cell-derived T_H_2 cell; NA, naïve; TG, transgenic; WT, wild-type.

Because unprimed MPT cells express higher Bcl6 levels than naïve CD4^+^ T cells (29), *Bcl6* expression levels in the MPT and MPT_H_2 cells of CNS2-GFP-TG mice with *Bcl6*-WT background were analyzed at rest (Figure 3A). *Bcl6* expression in GFP^+^ MPT and GFP^−^ MPT_H_2 cells was increased by sevenfold and threefold, respectively, compared with that in GFP^+^ MPT_H_2 cells. NAT_H_2 cells had markedly lower *Bcl6* expression than GFP^+^ MPT_H_2 cells. *Bcl6* expression in GFP^+^ MPT cells was slightly increased compared with that in GFP^−^ MPT cells. Consistent with the mRNA levels, Bcl6 protein expression was lower in GFP^+^ MPT_H_2 cells than in GFP^−^ MPT_H_2 cells (Figure 3B). Bcl6 protein levels in MPT cells from *Bcl6*-WT mice were higher than those in MPT_H_2 cells, whereas the protein levels in GFP^+^ MPT cells were slightly higher than those in GFP^−^ MPT cells. To address Bcl6 function, T_H_2 cytokine production by MPT_H_2 cells from *Bcl6*-WT-CNS2-GFP-TG mice was analyzed. T_H_2 cytokine protein (Figure 3C) and transcript levels (Figure 3D) were significantly greater in the GFP^+^ population than in the GFP^−^ population following stimulation, implying that Bcl6 function may be inhibited depending on its quantity and/or quality and that this inhibition may be involved in T_H_2 cytokine production in MPT_H_2 cells. Conversely, IFN-γ protein (Figure 3C) and transcript levels (Figure 3D) were undetectable and minimal, respectively, in both the GFP^+^ and GFP^−^ populations. Because Bcl6 binds to BSs (except BSIL13) (Figure 3E, top) and thereby reduces T_H_2 cytokine production in NAMT_H_2 cells (15), Bcl6 binding to each site in MPT_H_2 cells was analyzed by ChIP (Figure 3E, bottom). In *Bcl6*-WT and *Bcl6*-TG MPT_H_2 cells, Bcl6 binding was observed at all BS sites excluding BSIL13, BS1, and BS2. GFP^+^ cells had significantly less Bcl6 binding than GFP^−^ cells among *Bcl6*-WT and *Bcl6*-TG MPT_H_2 cells, whereas Bcl6 binding was augmented in *Bcl6*-TG MPT_H_2 cells. Thus, Bcl6 repressor functions may be regulated qualitatively (e.g., its binding ability) and quantitatively by its binding to T_H_2 cytokine gene foci. To investigate the effects of Bcl6 of STATs on histone modification in these foci, ChIP was performed for STAT5 and STAT6 binding to BSs and for histone H3 acetylation in MPT_H_2 cells (Figure 3F). STAT6 binding was marginal, whereas STAT5 binding was significantly decreased depending on Bcl6 levels, as indicated by attenuated histone acetylation.

### Bcl6 Represses *Il4* Expression by Binding to CNS2 in MPT_H_2 Cells

**Figure 4:**
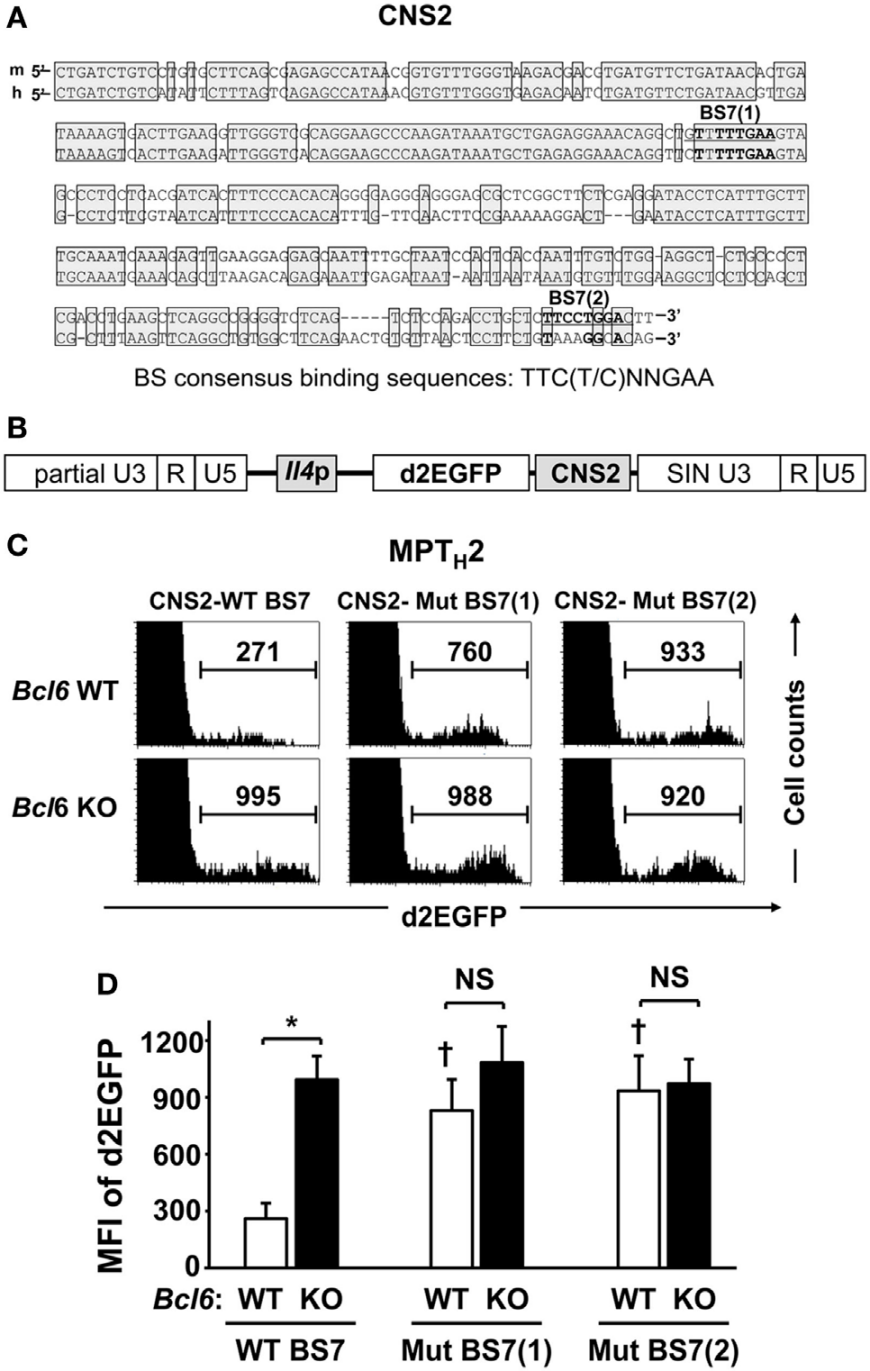
Role of Bcl6 in the CNS2 enhancer activity of MPT_H_2 cells. **(A)** A conserved sequence (positions +12805 to +13151 relative to the transcription start site; Mouse Genome Informatics accession no. 96556) in the CNS2 region of mice is shown with human CNS2, including BS7 (1) and (2). Conserved sequences between mice and humans are indicated by shaded boxes. **(B,C)** Splenic *Bcl6*-KO and *Bcl6*-WT MPT cells were cultured under T_H_2 conditions, and a retrovirus containing the *d2EGFP* reporter gene, with CNS2-WT BS7 **(B)**, CNS2-Mut BS7 (1), or CNS2-Mut BS7 (2), was introduced into T_H_2 cells on day 2 of culture. After 7 days of culture, cells were restimulated with anti-CD3 monoclonal antibodies for 8 h and subjected to FACS analysis of the intracellular MFI of d2EGFP. **(C)** Histograms of FACS analysis are representative of eight to nine independent experiments. Numbers in each column represent the MFI of d2EGFP. **(D)** Mean values of the MFI of d2EGFP are indicated. Data are presented as the mean ± SEM (*n* = 8–9). *P < 0.05, comparison between two groups is indicated; **^†^***P* < 0.05, compared with CNS2-WT. Bcl6, B-cell lymphoma 6; CNS, conserved noncoding sequence; d2EGFP, d2-enhanced green fluorescent protein; FACS, fluorescence-activated cell sorting; MFI, mean fluorescence intensity; MPT, memory phenotype CD4^+^ T; MPT_H_2 cell, MPT cell-derived T_H_2 cell; Mut, mutant; KO, knockout; WT, wild-type.

B-cell lymphoma 6, but not STAT proteins, binds to BS7 (Figures 3D,E) in the major *Il4* regulatory region. Although no significant Bcl6-mediated interaction was observed between BS7 in CNS2 regarding *Il4* regulation in NAMT_H_2 cells (15), CNS2 enhancer activity may be suppressed by Bcl6 through BS7 binding. FACS analysis indicated that GFP MFI levels related to CNS2 activation in MPT cells, including at two mutated sites, namely, BS7 (1) and (2) (Figure 4A), were inversely correlated with Bcl6 levels (Figure 1E). Therefore, the role of Bcl6 in enhancing activity in MPT_H_2 cells from *Bcl6*-WT or *Bcl6*-KO mice was investigated using a retrovirus reporter gene transfer vector (Figure 4B) designed to assess *Il4* promoter (p) activity by measuring the MFI for d2EGFP, a reporter protein, following stimulation with anti-CD3 and anti-CD28 mAbs (Figures 4C,D). Additionally, a CNS2 sequence containing WT or mutated BS7, that is, Mu-BS7 (1)-CNS2 and Mu-BS7 (2)-CNS2, were inserted downstream of *d2EGFP* (Figure 4B). The MFI for d2EGFP with CNS2-WT elements in *Bcl6*-KO cells was higher than that in *Bcl6*-WT cells. The MFI was augmented by mutations in both BS7 (1) and (2) in *Bcl6*-WT cells, whereas that of *Bcl6*-KO cells was not significantly changed (Figures 4C,D). Thus, Bcl6 mediated CNS2 suppression in MPT_H_2 cells and presumably in unprimed MPT cells.

### Bcl6 Represses *Il4* Expression by Binding to hcIE in MPT_H_2 Cells

**Figure 5:**
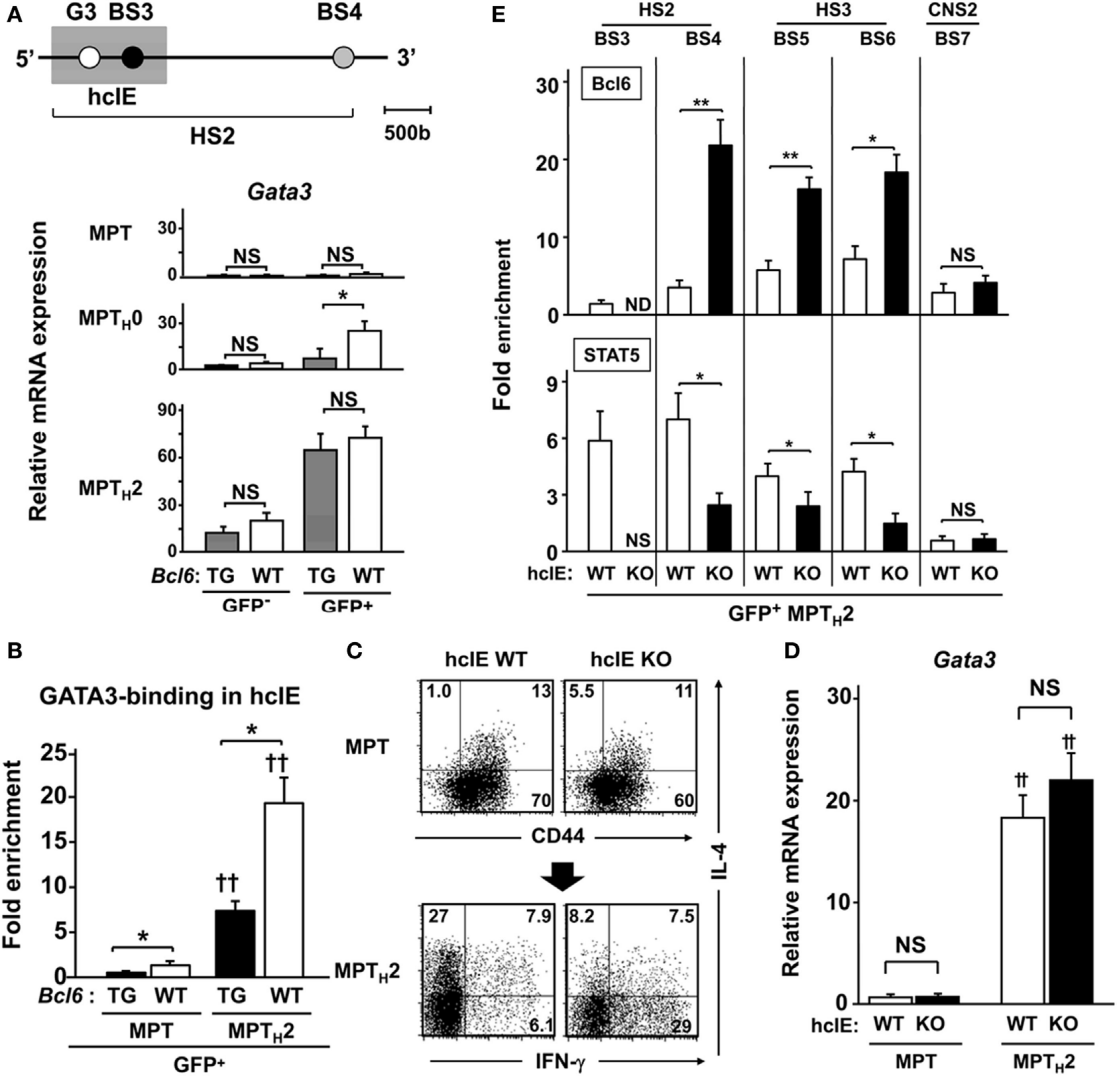
Role of Bcl6 in hcIE activity in MPT_H_2 cells. **(A*–*D)** KJ1-26^+^ cells among splenic MPT cells were cultured with ovalbumin peptides and antigen-presenting cells *in vitro* under T_H_0 or T_H_2 conditions. [**(A)** top] Diagram of the HS2 region in *Il4* intron 2, indicating regulatory regions. The shaded square indicates the hcIE region including the GATA3-binding site (G3) and BS3 within HS2. **(A)** *Gata3* mRNA levels in GFP^+^ and GFP^−^ MPT, MPT_H_0, and MPT_H_2 cells derived from *Bcl6*-TG and *Bcl6*-WT mice on a CNS2-GFP-TG background. **(B)** GATA3 binding to G3 analyzed by ChIP assays for GFP^+^ MPT and MPT_H_2 cells on a CNS2-GFP-TG background. **(C,D)** Analysis of splenic MPT cells or MPT_H_2 cells derived from hcIE-KO or hcIE-WT mice. **(A)** FACS analysis of intracellular cytokine populations of MPT cells by gating CD4^+^ CD49b^−^ T cells in the resting phase and MPT_H_2 cells restimulated with anti-CD3 monoclonal antibodies. The numbers in the corners represent the percentages among the gated T cells. **(D)** *Gata3* mRNA levels were measured by qRT-PCR for MPT and MPT_H_2 cells derived from hcIE-KO and hcIE-WT mice. **(E)** Bcl6 levels and STAT5 binding to each BS were analyzed by ChIP assay for GFP^+^ MPT_H_2 cells from hcIE-KO or hcIE-WT mice on a CNS2-GFP-TG background. All results are representative of three **(A,B)** or five **(C–E)** independent experiments with similar outcomes. Data are means ± SEMs (*n* = 9–10). *P < 0.05, comparison between two groups as indicated; **^†^***P* < 0.05, **^††^***P* < 0.01, compared with the MPT cells. Bcl6, B-cell lymphoma 6; BS, binding sequence; ChIP, chromatin immunoprecipitation; CNS, conserved noncoding sequence; FACS, fluorescence-activated cell sorting; hcIE, highly conserved intron enhancer; HS, DNase hypersensitive site; KO, knockout; MPT cell, memory phenotype CD4^+^ T cell; MPT_H_2 cell, MPT cell-derived T_H_2 cell; ND, not detected; NS, not significant; TG, transgenic; WT, wild-type.

Another *Il4* regulatory region, HS2 (1.2 kbp) located in intron 2, is a critical regulatory region for GATA3 binding-mediated *Il4* expression in NAT_H_2 cells in HS2-KO mice (33) (Figure 5A, top). A 222 bp DNA sequence of the hcIE region (Mouse Genome Informatics accession no. 5897323) (15) including BS3 and the GATA site (G3) in HS2 (Figure 5A, top) was studied. *Gata3* expression was low in unprimed MPT cells from *Bcl6*-WT and *Bcl6*-TG mice regardless of CNS2 activation, whereas MPT cells under the T_H_2 condition exhibited similar *Gata3* gene induction in *Bcl6*-WT and *Bcl6*-TG cells. Gene expression was augmented, particularly in GFP^+^ cells, and attenuated in a Bcl6-dependent manner in MPT_H_0 cells. However, further *Gata3* expression in MPT_H_2 cells was not significantly affected by Bcl6 levels (Figure 5A, bottom). We investigated the enhancer activity using a reporter construct, uncovering that Bcl6 inhibited hcIE function in MPT_H_2 cells (Figures S4A–C in Supplementary Material). Similarly, ChIP demonstrated that GATA3 binding to G3 was increased in CNS2-active GFP^+^ MPT_H_2 cells compared with that in unprimed GFP^+^ MPT cells and was significantly attenuated in *Bcl6*-TG background cells (Figure 5B). Thus, Bcl6 repressed *Il4* expression by downregulating GATA3-mediated hcIE activity in MPT_H_2 but not MPT cells.

To further examine the role of hcIE in T_H_2 cytokine production, we generated hcIE-KO mice and observed markedly diminished IL-4 production in hcIE-KO NAT_H_2 and NAMT_H_2 cells (15). Intracellular cytokine analysis revealed a similar frequency of IL-4^+^ populations in unprimed MPT cells in WT and hcIE-KO background mice, whereas IL-4^+^ MPT_H_2 cell development was impaired without changes in *Gata3* expression following hcIE deletion (Figures 5C,D). Bcl6 binding was augmented at BS4, BS5, and BS6 in intron 2 but not at CNS2 (BS7) in hcIE-KO MPT_H_2 cells compared with that in hcIE-WT background cells (Figure 5E), indicating that hcIE activity dampens Bcl6-mediated suppressor activity for intron 2 except at the CNS2 region.

### Bcl6 Suppresses Initial IL-4 Production in MPT Cells and T_H_2 Cell Differentiation

**Figure 6:**
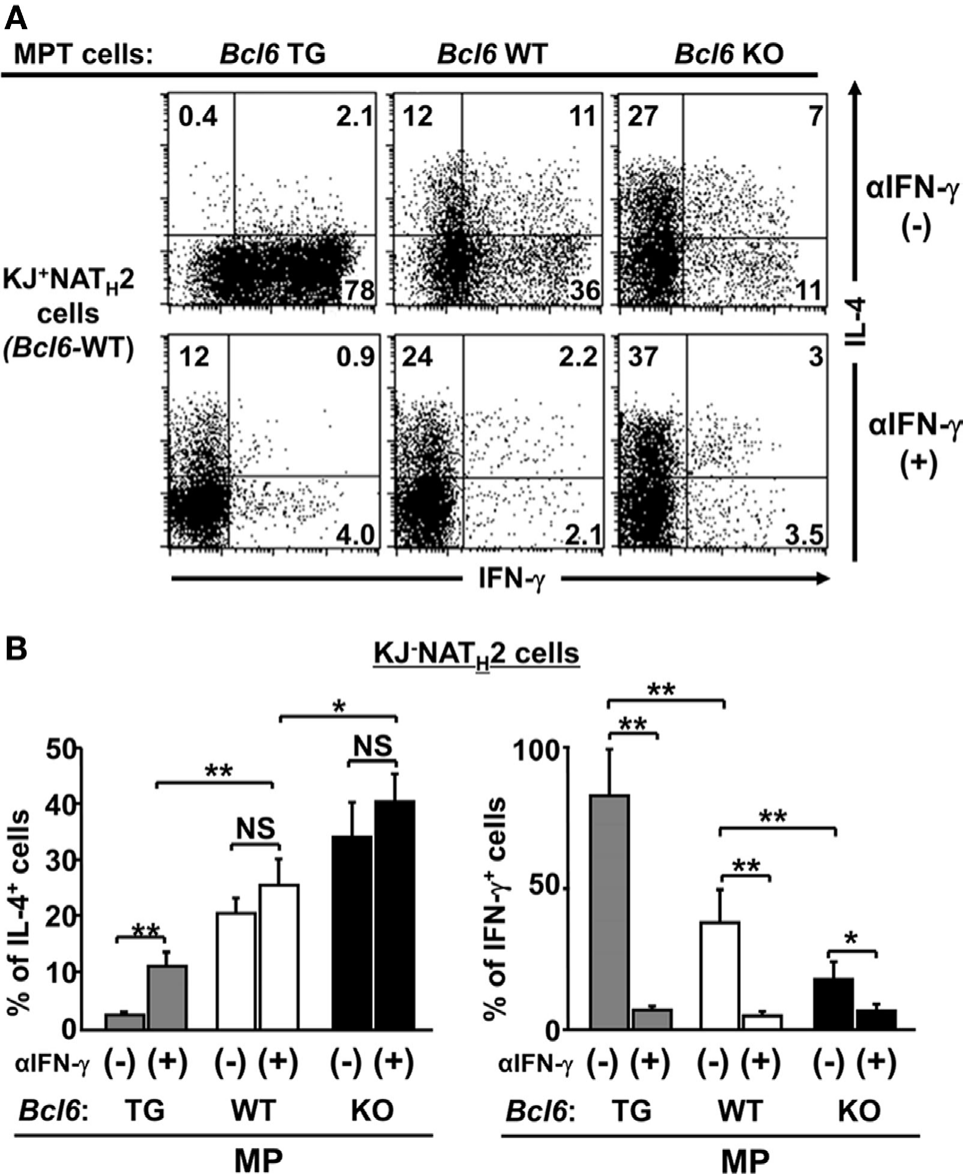
Role of Bcl6 in initial IL-4 production by MPT cells to induce NAT_H_2 cells *in vitro*. **(A,B)** *Bcl6*-WT KJ1-26^+^ naïve CD4^+^ T cells were cocultured with KJ1-26^−^ MPT cells (*Bcl6*-TG, *Bcl6*-WT, or *Bcl6*-KO) in the presence of soluble anti-CD3 and CD28 mAbs and irradiated CD11c^+^ DCs as the T_H_0 condition with or without anti-IFN-γ Abs. **(A)** FACS analysis of intracellular cytokines in each effector T cell type derived from KJ1-26^+^ naïve CD4^+^ T cells are presented as a representative figure among three independent experiments after restimulation with anti-CD3 mAbs. Numbers in the corners denote the percentages of gated KJ1-26^+^ CD4^+^ T cells. **(B)** Frequency of the populations of IL-4^+^ and IFN-γ^+^ KJ1-26^+^ T cells after reactivation. All results are representative of three independent experiments with similar outcomes. Data are presented as the mean ± SEM (*n* = 8–9). *P < 0.05; **P < 0.01. Ab, antibody; Bcl6, B-cell lymphoma 6; DC, dendritic cell; KO, knockout; mAb, monoclonal antibody; MPT cell, memory phenotype CD4^+^ T cell; NAT_H_2 cell; naïve CD4^+^ T cell-derived T_H_2 cell; TG, transgenic; WT, wild-type.

Because IL-4 production by MPT cells plays an important role in NAT_H_2 cell differentiation (28), to address the effects of Bcl6 on MPT cell function, *Bcl6*-WT-naïve KJ1-26^+^ CD4^+^ T cells were cocultured with KJ1-26^−^ MPT cells from *Bcl6*-TG, *Bcl6*-KO, or *Bcl6*-WT mice in the presence of soluble anti-CD3 and CD28 mAbs and irradiated CD11c^+^ DCs as the T_H_0 condition. Differentiation of IL-4-producing KJ1-26^+^ NAT_H_2 cells varied inversely with Bcl6 levels in KJ1-26^−^ MPT cells, whereas IFN-γ-producing NAT_H_1 cells differentiated in the opposite direction (Figures 6A,B). Because MP cell-derived IFN-γ may affect NAT_H_2 cell differentiation, we analyzed the T_H_2 skewing of naïve CD4^+^ T cells cocultured with MPT cells by excluding the effect of endogenous IFN-γ. Although T_H_2 skewing became prominent in the coculture in the presence of anti-IFN-γ Abs regardless of the Bcl6 genotype, the skewing was still suppressed in the presence of *Bcl6*-TG MPT cells. Therefore, Bcl6 plays an important role in suppressing MPT cell function to skew naïve CD4^+^ T cells toward the T_H_2 phenotype (Figures 6A,B). Furthermore, regardless of the *Bcl6* genotype, intrinsic IL-4 in MPT cells was involved in preserving the T_H_2 cell phenotype (Figures S5A,B in the Supplementary Material).

**Figure 7:**
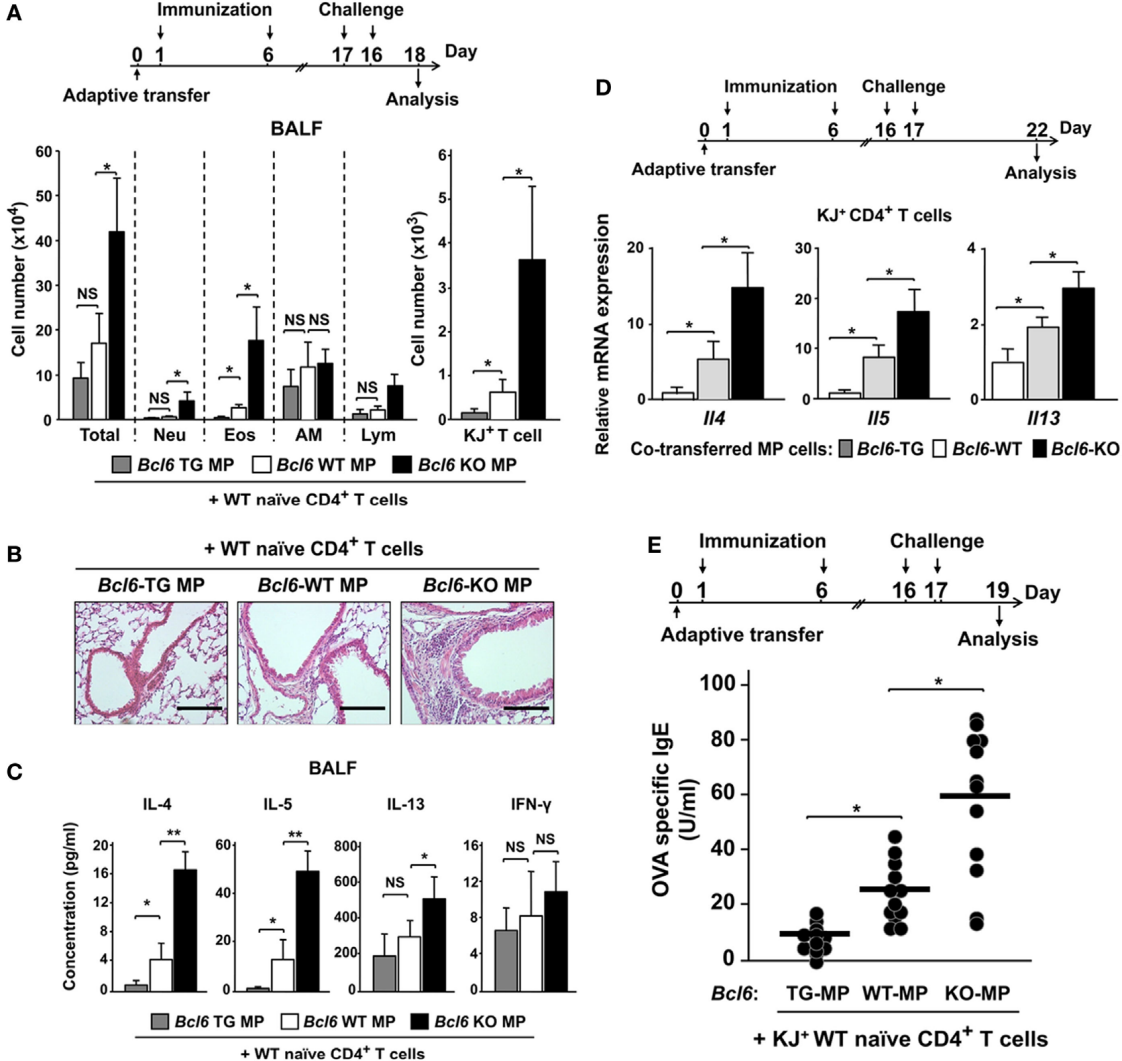
Role of Bcl6-mediated MPT cell functions in NAT_H_2 differentiation in an allergic murine model. [**(A)** top] Mixture of purified KJ1-26^−^ MPT cells (*Bcl6*-WT or *Bcl6*-KO) and KJ1-26^+^ WT naïve CD4^+^ T cells were transferred into BALB/c *nu/nu* mice intravenously (day 0). These mice were immunized with alum-conjugated OVA and then intratracheally challenged with OVA. [**(A)** bottom] Absolute cell numbers of Neu, Eos, AM, and Lym in BALF, **(B)** hematoxylin and eosin-stained, formalin-fixed lung sections (magnification: 200×), and **(C)** T_H_2 cytokine levels in the BALF of recipient mice 48 h after the last OVA challenge. **(D)** Relative *Il4, Il5*, and *Il13* expression mRNA in splenic KJ1-26^+^ T cells restimulated with anti-CD3 monoclonal antibodies 5 days after the last challenge. **(E)** OVA-specific IgE antibody titers in sera from each recipient of *Bcl6*-WT NAT_H_2 cells, plus MPT_H_2 cells transferred from *Bcl6*-TG, *Bcl6*-WT, or *Bcl6*-KO mice 2 days after the last challenge. All results are representative of four independent experiments with similar outcomes. Data are presented as the mean ± SEM (*n* = 5–7). *P < 0.05, **P < 0.01, comparison between two groups is indicated. AM, alveolar macrophages; BALF, bronchoalveolar lavage fluid; Bcl6, B-cell lymphoma 6; Eos, eosinophils; KJ^+^, KJ1-26-positive; KO, knockout; Lym, lymphocytes; MPT cell, memory phenotype CD4^+^ T cell; MPT_H_2 cell, MPT cell-derived T_H_2 cell; NAT_H_2 cell; naïve CD4^+^ T cell-derived T_H_2 cell; Neu, neutrophils; NS, not significant; OVA, ovalbumin; TG, transgenic; WT, wild-type.

As CNS2-active MPT cells are essential for inducing T_H_2 responses following immunization in an allergic murine model (28), we examined Bcl6 function in the MPT cell-induced response during the development of allergic immunity in BALB/c *nu/nu* mice undergoing adoptive transfer of *Bcl6*-WT-naïve CD4^+^ T cells (KJ1-26^+^) and MPT cells (KJ1-26^−^) from each respective *Bcl6* genotype. Following OVA challenge in the mice, the numbers of all inflammatory cells, neutrophils, eosinophils (left), and KJ1-26^+^ T cells (right) in whole lung tissues were significantly increased, being inversely correlated with Bcl6 levels in the transferred MPT cells (Figures 7A,B). In BALF from the recipients, the T_H_2 cytokine concentrations of IL-4, IL-5, and IL-13, but not IFN-γ, were decreased after the last OVA challenge, with this effect being dependent on Bcl6 levels in the transferred MPT cells (Figure 7C). In KJ1-26^+^ T cells (naïve-derived T_H_ cells) from the spleens of recipients after the last OVA challenge, T_H_2 cytokine mRNA expression (*Il4, Il5*, and *Il13*) was decreased depending on Bcl6 levels in the transferred MPT cells (Figure 7D). OVA-specific IgE levels in the sera were increased, in accordance with increased cytokine production after the last challenge (Figure 7E). This finding indicates that Bcl6 suppressed the development of allergic inflammation by reducing MPT cell function to facilitate NAT_H_2 cell differentiation.

### Bcl6 Attenuates the Synergistic Effect of MPT_H_2 Cells and NAM-LT_H_2 Cells on Allergic Responses

**Figure 8:**
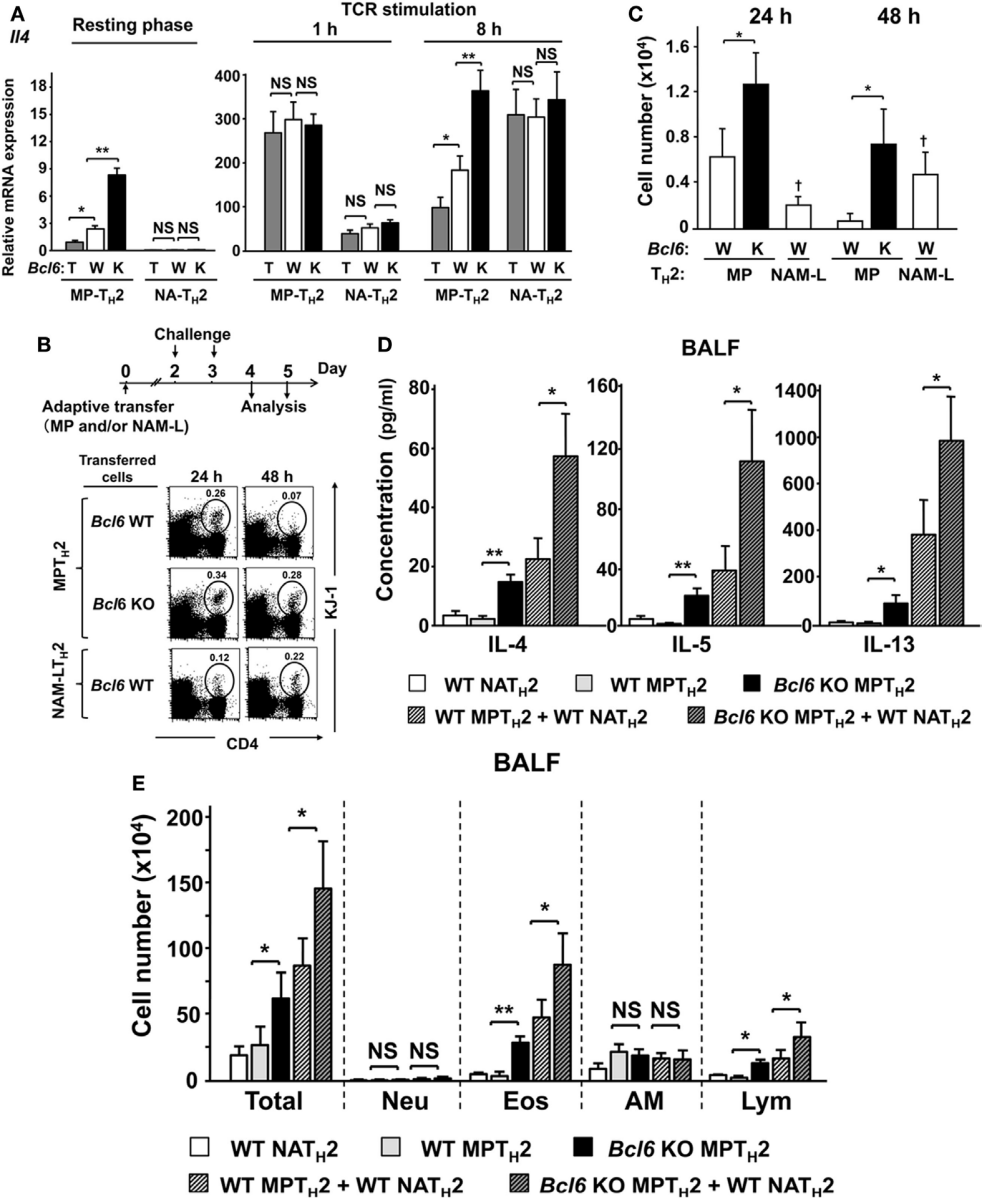
Role of Bcl6 in interactions between MPT_H_2 and naïve NAT_H_2 cells in allergy pathogenesis. **(A–E)** KJ1-26^+^ MPT_H_2 cells and NAT_H_2 cells were differentiated from the spleens of *Bcl6*-TG (T), *Bcl6*-WT (W), and *Bcl6*-KO (K) mice in the presence of OVA peptides and antigen-presenting cells in T_H_2 conditions. **(A)** *Il4* mRNA levels in each T_H_2 cell type were measured by qRT-PCR at rest and at 1 and 8 h after restimulation with anti-CD3 monoclonal antibodies. **(B–E)** *Bcl6*-WT BALB/c *nu/nu* mice were administered KJ1-26^+^ MPT_H_2 cells (3 × 10^7^), KJ1-26^+^ NAT_H_2 cells (3 × 10^7^), or combinations of MPT_H_2 (1.5 × 10^7^) and NAT_H_2 cells (1.5 × 10^7^) *via* adoptive transfer (day 0). **(B)** Representative FACS data for donor cells in circles with their percentages among total CD4^+^ T cells in whole lungs from recipients at 24 and 48 h after the last intratracheal OVA challenge. **(C)** Absolute numbers of KJ1-26^+^ cells in the lungs, **(D)** T_H_2 cytokine levels, and **(E)** cell types in the bronchoalveolar lavage fluid 48 h after the last challenge. All results are representative of four independent experiments with similar outcomes. Data are presented as the mean ± SEM (*n* = 8–10). *P < 0.05, **P < 0.01, comparison between two groups is indicated **(A,B,D,E)**; **^†^***P* < 0.05, compared with MPT_H_2 cells. AM, alveolar macrophages; Bcl6, B-cell lymphoma 6; Eos, eosinophils; FACS, fluorescence-activated cell sorting; KO, knockout; Lym, lymphocytes; MPT cell, memory phenotype CD4^+^ T cell; MPT_H_2 cell, MPT cell-derived T_H_2 cell; NAT_H_2 cell; naïve CD4^+^ T cell-derived T_H_2 cell; Neu, neutrophils; NS, not significant; OVA, ovalbumin; TCR, T cell receptor; TG, transgenic; WT, wild-type.

IL-4 levels were affected by Bcl6 in NAMT_H_2 cells, as previously reported (15). We focused on the functional difference in the spatiotemporal dynamics between MPT_H_2 and NAMT_H_2 cells. In the current study, NAM-LT_H_2 cells were analyzed as memory cells derived from naïve CD4^+^ T cells. In the resting phase, MPT_H_2 cells constitutively express *Il4*, the expression of which is reduced in a Bcl6-dependent manner. Following 1 h of restimulation, *Il4* expression in MPT_H_2 cells was increased to similar levels in each Bcl6 genotype, and the expression occurred earlier than that in *Bcl6*-WT-NAM-LT_H_2 cells. *Il4* expression levels were decreased in most MPT_H_2 cells, but not *Bcl6*-KO cells, in a Bcl6-dependent manner at 8 h after restimulation (Figure 8A). In NAM-LT_H_2 cells, *Il4* expression levels were low in the resting phase and increased after restimulation. The expression levels in *Bcl6*-WT-NAM-LT_H_2 cells were high, similar to those in *Bcl6*-KO MPT_H_2 cells at 8 h after restimulation (Figure 8A). The protein levels of IL-4 and IL-5, but not of IL-13, were consistent with the *Il4* expression pattern in each T_H_2 cell type (Figure S6 in Supplementary Material).

After adoptive transfer of each cell type (MPT_H_2 cells or NAM-LT_H_2 cells) with a DO11.10 genetic background into WT BALB/c *nu/nu* mice, cell migration into lung tissues following OVA antigen challenge was determined and presented as percentages (Figure 8B) and absolute cell numbers (Figure 8C). Among *Bcl6*-WT cells, MPT_H_2 cells had greater migratory capability compared with NAM-LT_H_2 cells at 24 h. The migration of MPT_H_2 cells decreased sequentially, whereas that of NAM-LT_H_2 cells increased at 48 h. The migration of *Bcl6*-KO MPT_H_2 cells was further augmented compared with that of *Bcl6*-WT cells. Next, we assessed the role of Bcl6 in interactions between MPT_H_2 and *Bcl6*-WT-NAM-LT_H_2 cells during allergic responses. WT BALB/c mice were adoptively transferred with combinations of each type of KJ1-26^+^ T_H_2 cells and sequentially challenged with OVA (Figures 8D,E). When *Bcl6*-WT-NAM-LT_H_2 or *Bcl6*-WT MPT_H_2 cells were transferred, T_H_2 cytokine levels (IL-4, IL-5, and IL-13) in the BALF were similar among recipients, whereas *Bcl6*-KO MPT_H_2 cells induced a fourfold to sevenfold increase in T_H_2 cytokine levels. Combined transfer of *Bcl6*-WT-NAM-LT_H_2 and *Bcl6*-WT MPT_H_2 cells resulted in synergistic cytokine production, which was further augmented when *Bcl6*-KO MPT_H_2 cells were transferred instead of *Bcl6*-WT MPT_H_2 cells (Figure 8D). The numbers of inflammatory cells, including eosinophils and lymphocytes, in the BALF (Figure 8E) were increased, in accordance with the increased production of cytokines, indicating that Bcl6 plays a critical role in regulating the functions of MPT_H_2 cells, which precede NAMT_H_2 cells in the development of local allergic pathology.

### IL-33 Reinforces IL-4 Production by MPT Cells

**Figure 9:**
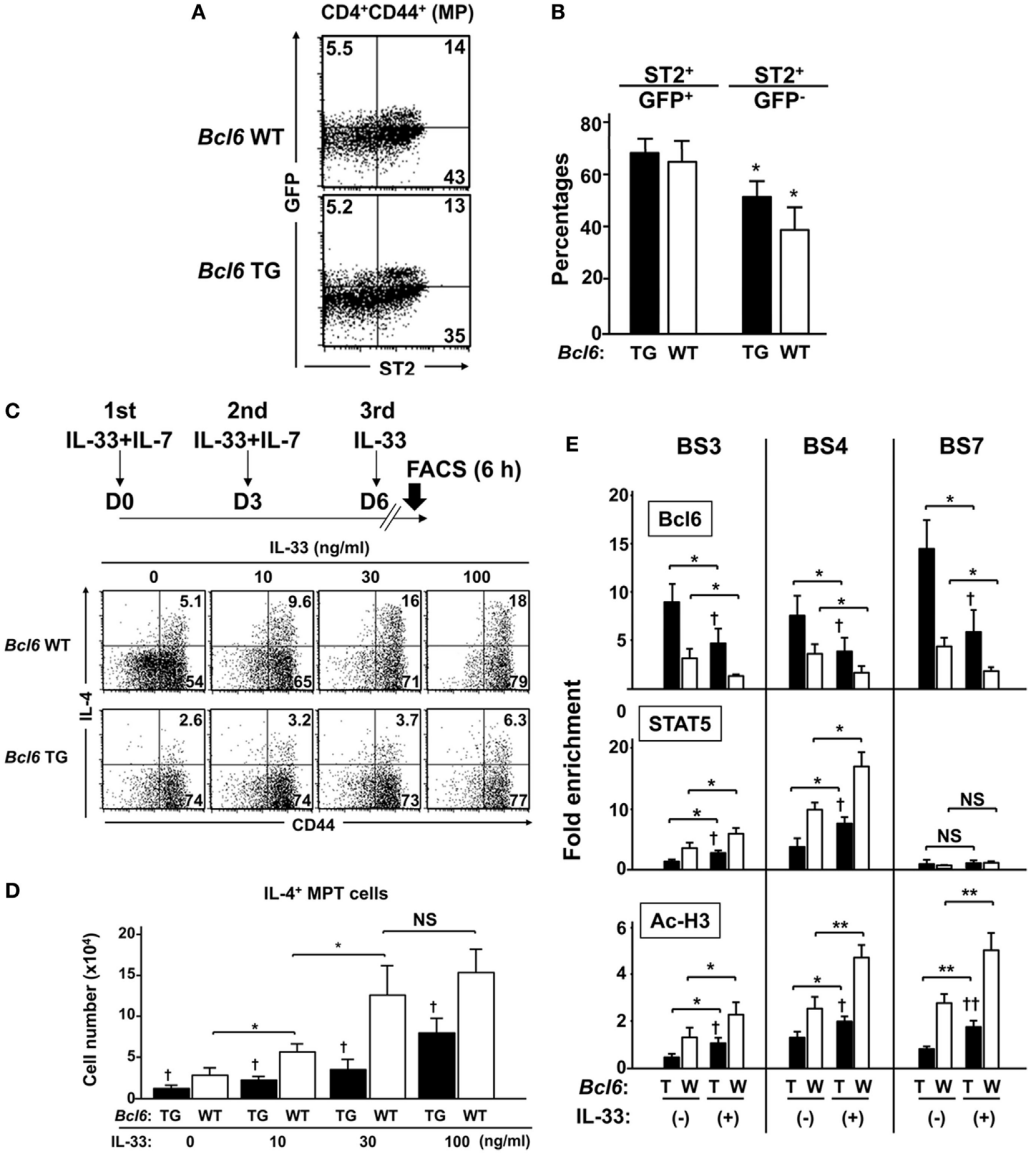
IL-33 reinforces IL-4 production by MPT cells through functional competition against the suppressor activity of Bcl6. **(A,B)** FACS analysis of splenic CNS2-GFP-TG MPT cells from *Bcl6-*TG and *Bcl6-*WT mice at rest. **(A)** Data show the expression of GFP and ST2 gated cells among all CD4^+^ CD44^+^ cells (representative of six independent experiments). **(B)** Percentages of ST2^+^ cells among GFP^+^ and GFP^−^ MPT cells. **(C,D)** IL-33 was added to the culture of MPT cells from *Bcl6-*TG and *Bcl6-*WT mice three times in the presence of IL-7. [**(C)** top] Six hours after the last IL-33 dose, MPT cells were analyzed for intracellular IL-4 levels. Numbers indicate the percentage of IL-4^+^ cells among all MPT cells. [**(C)** bottom] FACS analysis data are representative of four independent experiments. **(D)** Absolute numbers of IL-4^+^ MPT cells 8 h after the last IL-33 dose. **(E)** ChIP analysis of Bcl6 and STAT5 binding and Ac-H3 at each BS in CNS2-GFP^+^ MPT cells from *Bcl6-*TG (T) and *Bcl6-*WT mice(W). Cells were primed with or without IL-33 three times in the presence of IL-7. Analysis was performed 8 h after the last IL-33 dose. All results are representative of three **(A–D)** or four **(E)** independent experiments with similar outcomes. Data are presented as the mean ± SEM (*n* = 6–7). *P < 0.05, **P < 0.01, comparison between two groups is indicated*;* **^†^***P* < 0.05, **^††^***P* < 0.01, compared with WT. Ac-H3, acetylated histone H3; Bcl6, B-cell lymphoma 6; BS, binding sequence; ChIP, chromatin immunoprecipitation; CNS, conserved noncoding sequence; FACS, fluorescence-activated cell sorting; GFP, green fluorescent protein; MPT cell, memory phenotype CD4^+^ T cell; TG, transgenic; WT, wild-type.

Because we previously reported the effects of IL-33 on Bcl6-mediated histone modification in memory T_H_2 cells to augment IL-4 production (15), we focused in this study on the effect of IL-33 on MPT cells. FACS analysis demonstrated no significant difference in the cell-surface expression of ST2, an IL-33R subunit on MPT cells, between *Bcl6-*TG and *Bcl6*-WT mice (Figures 9A,B). ST2 was preferentially expressed on GFP^+^ MPT cells rather than GFP^−^ cells. When MPT cells were cultured in the presence of IL-7 for 6 days followed by IL-33 administration (Figure 9C, top), the frequency (Figure 9C) and absolute number (Figure 9D) of IL-4^+^ MPT cells increased in a concentration-dependent manner at 8 h following the last IL-33 dose. The effect of IL-33 on IL-4^+^ MPT cells was significantly reduced in *Bcl6-*TG cells compared with that in WT cells (Figures 9C,D). Consistent with the priming effect of IL-33, we observed elevated levels of histone acetylation at BS sites in the *Il4* locus with increased STAT5 histone association and decreased Bcl6 histone association. These effects of IL-33 on histone modification were attenuated in *Bcl6-*TG cells (Figure 9E).

## Discussion

The function of Bcl6 to regulated T_H_2 cytokine production is unclear. We found that Bcl6 negatively regulated IL-4 gene expression in MPT cells and their derived MPT_H_2 cells. Bcl6 inhibition significantly augmented IL-4 production by WT MPT_H_2 cells. Furthermore, IL-4 expression was reduced in T cell-specific *Bcl6-*TG MPT and *Bcl6-*TG MPT_H_2 cells, indicating a suppressive function of T cell-intrinsic Bcl6. CNS2 contains multiple putative binding sites for RBP-J, a critical modulator of notch signaling (34). CNS2 is regulated by notch signals to control initial IL-4 expression in MPT cells (28). We demonstrated that Bcl6 binds to CNS2, leading to suppression of its enhancer activity in MPT_H_2 cells. Bcl6 antagonizes notch-dependent transcription (35, 36). However, *Rbpj* deletion does not alter epigenetic markers on the CNS2 site in T_FH_ cells (29). Thus, to elucidate the positive regulatory mechanism of the activation of CNS2, a target of Bcl6 in MPT cells, further analysis is required.

GATA3 binding in the HS2 enhancer region is critical for NAT_H_2 (15, 35) and NAMT_H_2 cells (15). However, extremely low GATA3 expression might not be associated with IL-4 production in MPT cells. We demonstrated that GATA3-mediated hcIE activation is not essential for IL-4 production by MPT cells (Figures 5B–D). However, MPT_H_2 cell differentiation requires hcIE enhancer activity, which induces permissive histone modification of the *Il4* locus by cooperating with STAT5 and GATA3 (37). Bcl6 directly bound to and interfered with hcIE function in MPT_H_2 cells. Accordingly, we suggest that diverse Bcl6 functions regulate IL-4 production in MPT_H_2 and MPT cells. The locus control region (LCR) at the Rad50 gene is also extremely important for T_H_2 cytokine expression. This region is considered to be involved in coordinating T_H_2 cytokine genes including IL-4. We previously reported the GATA3-binding site and Bcl6/STAT-binding sites in conserved regions (T_H_2LCR) in the Rad50 gene in another study (15). We also reported that Bcl6 binding in the LCR is augmented by disruption of hcIE in *Il4*, indicating that Bcl6-mediated T_H_2LCR organizes T_H_2 cytokine gene including IL-4. Therefore, T_H_2LCR may be implicated in *Il4* regulation in CNS2-active MPT cells. To elucidate the role of T_H_2LCR, further studies using region-deficient mice are required.

B-cell lymphoma 6 has various regulatory functions associated with cell viability and cytokine production, although the detailed molecular mechanisms have not been clarified. We observed that CNS2-active MPT cells contained high Bcl6 levels that declined following augmented IL-4 production under T_H_2 priming conditions. Intriguingly, in *Bcl6*-WT MPT_H_2 cells, the CNS2-active population exhibited markedly lower *Bcl6* levels and higher *Il4* levels than the CNS2-inactive population. Greater Bcl6 mRNA levels in CNS2-active MPT cells than in the CNS2-inactive population in WT mice have been reported (29), whereas we observed slight differences in expression between these two populations. However, Bcl6 protein levels in CNS2-active *Bcl6*-WT MPT cells were inversely decreased relative to those in the CNS2-inactive MPT cells. Therefore, when pleiotropic Bcl6 effects are required in the same cellular environment, its function may be quantitatively controlled at transcriptional, translational, or post-transcriptional levels.

We previously demonstrated that T_H_2 cytokine genes are negatively regulated by Bcl6 through chromatin remodeling and that interactions between Bcl6 and STAT5 are physiologically implicated in histone modulation and consequently cytokine production in NAMT_H_2 cells rather than NAT_H_2 cell differentiation (15). In a previous report, we advocated that STAT5 and GATA3 cooperate in permissive histone modification of the *Il4* locus by binding to hcIE and that STAT5- and GATA3-mediated epigenetic activity of hcIE may be controlled by directly and/or indirectly preventing the Bcl6-mediated silencing. In addition, Bcl6 binding to BS4, BS5, and BS6 in the *Il4* locus was augmented upon hcIE disruption in differentiating T_H_2 cells. Therefore, even in the presence of high levels of Bcl6, *Bcl6*-TG naïve CD4^+^ T cells could differentiate into T_H_2 cells under the T_H_2 full commitment condition. Conversely, when naïve *Bcl6*-TG, *Bcl6*-WT, and *Bcl6*-KO CD4^+^ T cells are stimulated under the T_H_0 condition, IL-4 production by restimulated CD4^+^ T cells was reduced in a Bcl6 level-dependent manner. Therefore, we propose that the repressor activity of Bcl6 in the *Il4* locus including hcIE and CNS2 can be determined in functional balance with transcriptional activators, such as GATA3, STATs, and RBP-J, in both MPT_H_2 and NAT_H_2 cells. Accordingly, both quantitative and qualitative Bcl6 functional modifications, such as reduced binding activity (15), may be implicated in the gene regulation of *Il4*. Notably, we observed that Bcl6 binding to the *Il4* locus is higher in CNS2-GFP^−^ *Bcl6*-TG MPT_H_2 cells than in GFP^+^ B*cl6*-TG cells. Because enhancers can generally regulate transcription by interacting with enhancers or promoters *via* chromatin looping mechanisms, we propose that CNS2 may also stimulate *Il4* transcription *via* physical interactions with hcIE, which may influence and organize Bcl6/STAT binding in hcIE. Therefore, Bcl6 binding to the *Il4* locus may exceed STAT5 binding *via* Bcl6-mediated inhibition of CNS2 activity.

In earlier reports, we and other groups uncovered that Bcl6 has no significant intrinsic function in the differentiation of naïve CD4^+^ T cells into T_H_1/T_H_2 cells in full commitment experiments *in vitro*. In later studies focusing on T_FH_ cells, Bcl6 suppressed effector T cells, including T_H_1, T_H_2, and T_H_17 cells, resulting in the induction of T_FH_ cell differentiation. The current study indicated that Bcl6 promotes IFN-γ production *via* by inhibiting IL-4 production in activated naïve CD4^+^ T cells and MPT cells in some experimental settings, rather than inhibiting IL-4 production by promoting IFN-γ production.

Contrarily, we previously reported that Bcl6 plays an important anti-apoptotic role in effector-derived memory precursor CD4^+^ T cells, suggesting that Bcl6 is involved in long-term memory T cell survival (17, 30, 38). We observed that the numbers of splenic MPT cells and, intriguingly, CNS2-active GFP^+^ MPT cells were positively associated with intrinsic Bcl6 levels, whereas the MFI of GFP was reduced in *Bcl6*-TG cells. Recently, CNS2-active GFP^+^ CD4^+^ T cells in secondary lymphoid tissues were found to have a high *Bcl6* expression phenotype, similar to T_FH_ cells (29). Bcl6 is a master regulatory factor for T_FH_ cell differentiation. However, a substantial *Bcl6*-KO MPT cell population exists, and we suggested that CNS2-active MPT cells are not necessary as part of the T_FH_ cell lineage. Although the molecular mechanism is unclear, Bcl6 may be implicated in, but not essential for, the development and/or maintenance of MPT and MPT_H_2 cells.

NAMT_H_2 cells have an important role in chronic allergic responses (15), although the relationship between NAMT_H_2 and MPT_H_2 cells is unclear. We observed that T_H_2 cytokine production peaked and declined earlier in *Bcl6*-WT-MPT_H_2 cells than in WT-NAM-LT_H_2 cells. Moreover, the migratory function of MPT_H_2 cells was superior to that of NAM-LT_H_2 cells, albeit due to an unknown mechanism. Because CNS2 and *Il4* are constitutively activated in MPT_H_2 cells but not in NAMT_H_2 cells (15), MPT_H_2 cells might influence NAMT_H_2 cell function in chronic allergy. Accordingly, MPT_H_2 cells organize T_H_2 immune responses directly and/or indirectly by regulating NAMT_H_2 cell function, resulting in allergy enhancement.

IL-4 production by CNS2-active MPT cells induced T_H_2 responses by inducing the differentiation of NAT_H_2 cells from naïve CD4^+^ T cells and their self-differentiation into MPT_H_2 cells following immunization (28). We confirmed initial IL-4 production from MPT cells in this study. Because CNS2-active MPT cells do not belong to the T_FH_ cell lineage derived from naïve CD4^+^ T cells (29) but they rather develop from selected thymocytes among those expressing other MHC class II markers (39), IL-4^+^ MPT cells might develop independently of naïve CD4^+^ T cells during thymic differentiation. In that case, sequentially differentiated MPT_H_2 cells as well as MPT cells contribute to the early pathology of some allergies.

When considering the nature of Bcl6 in MPT and MPT_H_2 cells in pathologic conditions, we should determine whether *Bcl6* expression can be modified without artificial gene manipulation at both protein and RNA levels. Recently, we reported that a T_H_2-promoting factor, namely, IL-33-mediated breakdown of Bcl6 in NAMT_H_2 cells, is likely involved in allergies (15) given the effect of IL-33 on both MPT and NAMT_H_2 cells. Therefore, the IL-33/Bcl6 axis might participate in allergy pathology *via* the regulation of *Il4* in MPT cells to promote disease development in MPT_H_2 and NAMT_H_2 cells, contributing to the maintenance and exacerbation of disease pathology.

In summary, the current study provides evidence for a novel role of Bcl6 in the functional regulation of MPT and MPT_H_2 cells, implying interplay between Bcl6 and transcriptional activators to promote the production of relevant T_H_2 cytokines, particularly IL-4. Thus, T_H_2 cell-promoting factors that suppress Bcl6 function may represent crucial therapeutic targets for T_H_2 cell-mediated diseases.

## Ethics Statement

This study was carried out in accordance with the recommendations of the Chiba University Resolution on Use of Animals in Research. The protocol was approved by the Institutional Animal Care and Use Committee at Chiba University School of Medicine. The mice were maintained under specific pathogen-free conditions in the animal center of Chiba University Graduate School of Medicine.

## Author Contributions

MA and TO jointly designed the experiments and directed the study and wrote the manuscript. MA, TO, YK, JI, TT, NT, HW-T, LF, AS, HH, and MH performed the experiments. MA, TO, MH, YF, and KK analyzed the data and generated the figures. YF, KT, TT, and TF provided reagents and/or support for the analysis.

## Conflict of Interest Statement

The authors declare that the research was conducted in the absence of any commercial or financial relationships that could be construed as a potential conflict of interest.

## Abbreviations

Abs, antibodies; APC, antigen-presenting cell; BAL, Bronchoalveolar lavage; BALF, Bronchoalveolar lavage fluid; Bcl6, B-cell lymphoma 6; BS, binding sequence; ChIP, chromatin immunoprecipitation; CNS, conserved noncoding sequence; DC, dendritic cell; EGFP, enhanced green fluorescent protein; FACS, fluorescence-activated cell sorting; GFP, green fluorescent protein; hcIE, highly conserved intron enhancer; HS, DNase hypersensitive site; KO, knockout; LCR, locus control region; LTR, long terminal repeat; mAbs, monoclonal antibodies; MFI, mean fluorescence intensity; MPT cell, memory phenotype CD4^+^ T cell; MPT_H_2 cell, MPT cell-derived T_H_2 cell; NAM-LT_H_2 cell, NAT_H_2 cell-derived memory-like T_H_2 cell; NAMT_H_2 cell, NAT_H_2 cell-derived memory cell; NAT_H_2 cell, naïve CD4^+^ T cell-derived T_H_2 cell; OVA, ovalbumin; PCR, polymerase chain reaction; STAT, signal transducer and activator of transcription; TCR, T cell receptor; T_FH_ cell, T follicular helper cell; TG, transgenic; WT, wild-type.
