# Supplementary material for: Allergic TH2 Response Governed by B-Cell Lymphoma 6 Function in Naturally Occurring Memory Phenotype CD4+ T Cells
Source: Front Immunol. 2018 Apr 10;9:750. doi: 10.3389/fimmu.2018.00750 (PMC5904433; doi:10.3389/fimmu.2018.00750)
Supplement: Supplementary file 1 [file Presentation_1.PDF]

Supplement

Allergic T_H2 response governed by Bcl6 function in naturally occurring memory phenotype $CD4^+$ T cells

Authors:

Takashi Ogasawara[1], Yuko Kohashi[2], Jun Ikari[1], Toshibumi Taniguchi[2], Nobuhide Tsuruoka[3], Haruko Watanabe-Takano[2], Lisa Fujimura[4], Akemi Sakamoto[2], Masahiko Hatano[2], Hirokuni Hirata[5], Yasutsugu Fukushima[5], Takeshi Fukuda[6], Kazuhiro Kurasawa[7], Koichiro Tatsumi[1], Takeshi Tokuhisa[8], and Masafumi Arima[2,7*]

[1]Department of Respirology (B2), [2]Department of Biomedical Science (M14), and [3]Departments of Reproductive Medicine (G4), Chiba University Graduate School of Medicine, Chiba, Japan

[4]Biomedical Research Center, Chiba University, Chiba, Japan

[5]Department of Respiratory Medicine and Clinical Immunology, Dokkyo Medical University Koshigaya Hospital, Koshigaya, Japan

[6]Department of Pulmonary Medicine and Clinical Immunology and [7]Department of Rheumatology, Dokkyo Medical University School of Medicine, Mibu, Japan

[8]Department of Developmental Genetics, Chiba University Graduate School of Medicine, Chiba, Japan.

Results

Regulatory role of Bcl6 in the CNS2-active MPT cells in the T_H condition

We analyzed the effects of Bcl6 on the maintenance of CNS2-GFP^+ MPT cells in each culture setting. Regarding the maintenance of GFP^+ cells, a promoting effect of the T_H2 condition and inhibitory effect of T_H1 condition were observed regardless of the *Bcl6* genotype (Figure S1, *A*), whereas excess amounts of Bcl6 significantly reduced GFP^+ cell maintenance under the T_H0 condition (Figure S1, *A*, *right*). The MFI of GFP was significantly decreased in both WT and TG cells under T_H0 and T_H1 conditions and increased in WT cells under the T_H2 condition (Figure S1, *B*). The MFI was significantly reduced in *Bcl6*-TG cells compared with that in WT cells under T_H0 and T_H2 conditions (Figure S1, *B*). Therefore, Bcl6 appears to function as a suppressor for CNS2 activity.

Effect of Bcl6 on the initial IL-4 production by naive CD4$^+$ T cells

We addressed the effect by culture of naive *Bcl6*-TG, *Bcl6*-WT, and *Bcl6*-KO CD4$^+$ T cells under the T_H0 condition with or without anti-IFN-γ Abs (Figure S2). The results illustrated that even in the presence of anti-IFN-γ Abs, IL-4 production by restimulated CD4$^+$ T cells was reduced in proportion to the Bcl6 level. Therefore, Bcl6 could suppress the initial IL-4 production by naive CD4$^+$ T cells under the T_H0 condition even when the effects of IFN-γ are blocked.

Interactive activation between MPT cells with different intrinsic Bcl6 levels

We analyzed mixed cultures of *Bcl6*-WT MPT cells with either *Bcl6*-TG or *Bcl6*-KO cells under the T_H0 condition and examined cytokine production by the restimulated *Bcl6*-WT MPT cells (Figure S3). Because the background of the *bcl6*-manipulated mice was DO11.10 BALB/c, KJ1-26$^+$ MPT cells (*Bcl6*-WT) were analyzed for T_H skewing in the presence of KJ1-26$^-$ MPT cells (*Bcl6*-WT, TG, or KO). When *Bcl6*-WT MPT cells (KJ1-26$^+$) were cocultured with *Bcl6*-TG MPT cells, T_H2 and T_H1 cell skewing were slightly inhibited and augmented, respectively. Conversely, when cocultured with *Bcl6*-KO MPT cells, WT MPT cells were skewed clearly toward T_H2 cells with reduced T_H1 skewing. These results indicate that increased IL-4 production in *Bck6*-KO MP cells autoaccelerate T_H2 cell differentiation by preventing T_H1 cell differentiation. Thus, Bcl6 appears to promote IFN-γ production by inhibiting IL-4 production rather than inhibiting IL-4 production via the promotion of IFN-γ production.

Role of Bcl6 in hcIE activity in MPT$_H$2 cells

We investigated the enhancing activity of Bcl6 using a reporter construct (*Il4*p-d2EGFP-hcIE) (Fig S1, *A*). The MFI levels of *d2EGFP* in *Bcl6*-KO MPT_H2 cells were higher than those of *Bcl6*-WT cells. Mutation of BS3 (MutBS3) and a GATA3 site (MutG3) in hcIE significantly reduced MFIs in *Bcl6*-WT and *Bcl6*-KO cells, and MFIs in the presence of hcIE-MutG3 elements were greater in *Bcl6*-KO cells than in WT cells (Fig S4, *B* and *C*). Although Bcl6 and STATs appeared to mutually bind to BS3 in hcIE (Figure S4, *E* and *F*) and the precise role of the BS3 region is unclear, BS3 is presumed to have an enhancer function.

Role of Bcl6 in the initial IL-4 production by MPT cells to induce NAT$_H$2 cells *in vitro*

To investigate a role of intrinsic IL-4 in preserving the self-TH2 cell phenotype, we analyzed the effect of IL-4 neutralization on IL-4 production and its gene expression by activated MPT cells. Regardless of the *Bcl6* genotype, IL-4 production was significantly inhibited in the presence of anti-IL-4 Abs, indicating that the maintenance of the T_H2 cell phenotype depended on IL-4 self-production (Figure S5, *A* and *B*).

Role of Bcl6 in interactions between MPT$_H$2 and NAT$_H$2 cells for allergy pathogenesis

The protein levels of IL-4 and IL-5, but not IL-13, produced by each T_H2 cell type were consistent with the pattern of *Il4* expression. Namely, the protein levels of IL-4 and IL-5, but not IL-13, in MPT$_H$2 cells were reduced in the presence of Bcl6 in a concentration-dependent manner from 24 to 48 h after restimulation. The levels of T_H2 cytokine production by *Bcl6*-WT-NAM-LT$_H$2 cells as compared with those by MPT$_H$2 cells were lower at 24 h after restimulation and greater at 48 h (Figure S6).

Figure legends

Figure S1. Regulatory role of Bcl6 in CNS2-active MPT cells in the T_H condition. KJ1-26^+ MPT cells among splenocytes from *Bcl6*-TG and *Bcl6*-WT DO11.10 mice were cultured with ovalbumin peptides and antigen-presenting cells *in vitro* for 7 days to produce T_H0, T_H1, or T_H2 cells. Uncultured cells and cultivated cells were restimulated with anti-CD3 monoclonal antibodies. After 8 h, CNS2 activation-related GFP^+ cells among gated KJ1-26^+ $CD4^+$ T cells were analyzed by FACS. **(A,** left**)** The numbers in the corners denote the percentages of gated T cells. **(A**, right**)** Frequency of GFP^+ MPT cells. **(B)** MFI of GFP in MPT cells. Data are presented as the mean ± SEM (n = 3). *P < 0.05; **P < 0.01, comparison between two groups as indicated. $^{\dagger}P$ < 0.05, compared with uncultured cells. The presented data are representative of three independent experiments. Bcl6, B-cell lymphoma 6; CNS, conserved noncoding sequence; FACS, fluorescence-activated cell sorting; GFP, green fluorescent protein; MPT cell, memory phenotype $CD4^+$ T cell; MFI, mean fluorescence intensity; NS, not significant; TG, transgenic; WT, wild-type.

Figure S2. Effect of Bcl6 on the initial IL-4 production by naive $CD4^+$ T cells. KJ1-26^+ naïve $CD4^+$ T cells among splenocytes from *Bcl6*-TG, *Bcl6*-WT, and *Bcl6*-KO DO11.10 mice were cultured with ovalbumin peptides and antigen-presenting cells *in vitro* for

7 days under T_H0 condition with (**top**) or without (**bottom**) anti-IFN-γ antibodies. Cells were restimulated with anti-CD3 monoclonal antibodies. After 8 h, IL-4- and IFN-γ-producing cells among gated KJ1-26$^+$ CD4$^+$ T cells were analyzed via FACS. Data presented are representative of three independent experiments. Numbers in the corners represent percentages among gated T cells. Bcl6, B-cell lymphoma 6; FACS, fluorescence-activated cell sorting; KO, knockout; MPT cell, memory phenotype CD4$^+$ T cell; TG, transgenic; WT, wild-type.

Figure S3. Interactive activation between MPT cells with different intrinsic Bcl6 levels. *Bcl6*-WT KJ1-26$^+$ MPT cells were cocultured with KJ1-26$^-$ MPT cells (*Bcl6*-TG, *Bcl6*-WT, or *Bcl6*-KO) in the presence of soluble anti-CD3 and CD28 mAbs and irradiated CD11c$^+$ DCs as the T_H0 condition. FACS analysis of intracellular cytokines in each effector T cell type derived from KJ1-26$^+$ MPT cells are presented as a representative figure of three independent experiments after restimulation with anti-CD3 monoclonal antibodies. Numbers in the corners represent the percentages of gated KJ1-26$^+$ CD4$^+$ T cells. Bcl6, B-cell lymphoma 6; FACS, fluorescence-activated cell sorting; KO, knockout; mAb, monoclonal antibody; MPT cell, memory phenotype CD4$^+$ T cell; MPT_H2 cell, MPT cell-derived T_H2 cell; TG, transgenic; WT, wild-type.

Figure S4. Role of Bcl6 in hcIE activity in MPT_H2 cells. **(A)** The murine hcIE region (positions +1180 to +1401 relative to the transcription start site) was included in reporter constructs. **(B**, **C)** Splenic *Bcl6*-KO and *Bcl6*-WT MPT cells were cultured under T_H2 conditions, and a retrovirus containing *d2EGFP* and hcIE-WT BS3/G3, hcIE-Mut BS3, or hcIE-Mut G3 was introduced into cultivated MPT_H2 cells. After culture for 7 days, the cells were restimulated with anti-CD3 mAbs and subjected to FACS analysis to measure intracellular d2EGFP MFIs. **(B)** The number in each column represents the MFI. **(C)** Histograms of FACS analysis from seven independent experiments. All results are representative of seven independent experiments with similar outcomes. Data are presented as the mean ± SEM (n = 7–8). *$P < 0.05$, comparison between two groups is indicated; $^{\dagger}P < 0.05$, compared with hcIE-WT BS3/G3. Bcl6, B-cell lymphoma 6; d2EGFP, d2-enhanced green fluorescent protein; FACS, fluorescence-activated cell sorting; hcIE, highly conserved intron enhancer; KO, knockout; MFI, mean fluorescence intensity; MPT cell, memory phenotype CD4$^+$ T cell; MPT_H2 cell, MPT cell-derived T_H2 cell; WT, wild-type.

Figure S5. Role of Bcl6 in initial IL-4 production by MPT cells to induce NAT_H2 cells

in vitro. MPT cells from *Bcl6*-TG, *Bcl6*-WT, or *Bcl6*-KO spleens were activated with anti-CD3 and anti-CD28 mAbs under the $\mathrm{T_H}$0 condition in the presence of anti-IL-4 neutralizing Abs. **(A)** FACS analysis data of intracellular cytokines in each effector T cell type derived from MPT cells represent the findings of six independent experiments after restimulation with anti-CD3 mAbs. Numbers in the corners denote the percentage among gated CD44^{high} CD4^{+} T cells. **(B)** Amounts of *Il4* mRNA in each effector cell type were measured by qRT-PCR at rest and after restimulation. All results are representative of three independent experiments with similar outcomes. Data are presented as the mean ± SEM (n = 6–8). $^{**}P < 0.01$, comparison between two groups is indicated. Ab, antibody; Bcl6, B-cell lymphoma 6; FACS, fluorescence-activated cell sorting; KO, knockout; mAb, monoclonal antibody; MPT cell, memory phenotype CD4^{+} T cell; NA$\mathrm{T_H}$2 cell; naïve CD4^{+} T cell-derived $\mathrm{T_H}$2 cell; TG, transgenic; WT, wild-type.

Figure S6. Role of Bcl6 in interactions between MP$\mathrm{T_H}$2 and NA$\mathrm{T_H}$2 cells in allergy pathogenesis. KJ1-26^{+} MP$\mathrm{T_H}$2 (MP) and NAM-L$\mathrm{T_H}$2 (NAM-L) cells were differentiated from the spleens of *Bcl6*-TG (T), *Bcl6*-WT (W), and *Bcl6*-KO (K) mice in the presence of OVA peptides and APCs *in vitro* for 7 days under the $\mathrm{T_H}$2 condition. Amounts of $\mathrm{T_H}$2 cytokines in the culture supernatants of each $\mathrm{T_H}$2 cell type at 24 and 48 h after restimulation. All results are representative of three independent experiments with similar outcomes. Data are presented as the mean ± SEM (n = 6–7). $^{*}P < 0.05$, $^{**}P < 0.01$, comparison between two groups is indicated; $^{\dagger}P < 0.05$, compared with *Bcl6*-WT MP$\mathrm{T_H}$2 cells. NS, not significant; APC, antigen-presenting cell; Bcl6, B-cell lymphoma 6; KO, knockout; MPT cell, memory phenotype CD4^{+} T cell; MP$\mathrm{T_H}$2 cell, MPT cell-derived $\mathrm{T_H}$2 cell; NA$\mathrm{T_H}$2 cell, naïve CD4^{+} T cell-derived $\mathrm{T_H}$2 cell; OVA, ovalbumin; TG, transgenic; WT, wild-type.

Fig S1

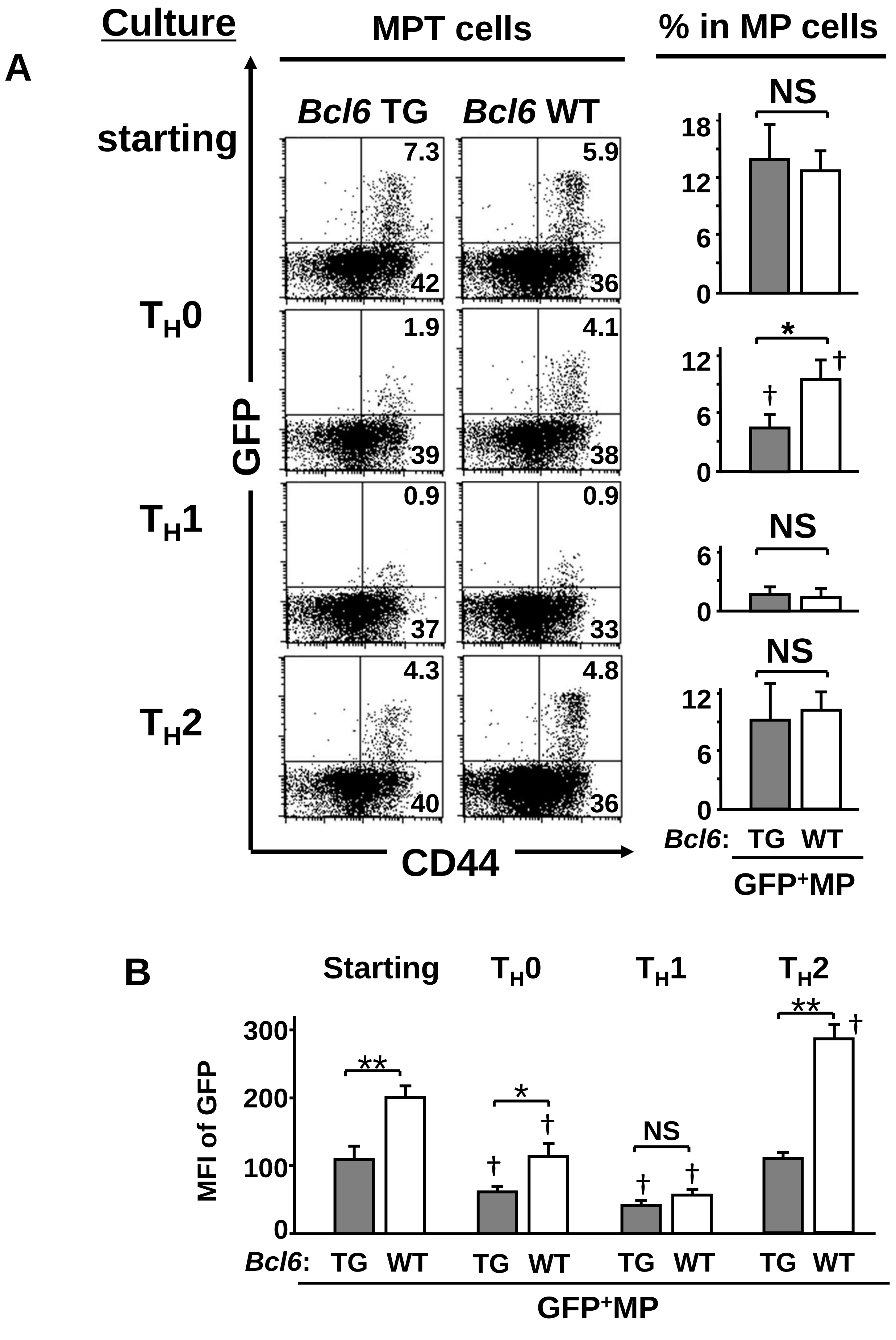
A
Culture
MPT cells
% in MP cells
Bcl6 TG
Bcl6 WT
starting
7.3
42
5.9
36
NS
18
12
6
0
TH0
1.9
39
4.1
38
*
12
6
0
†
†
TH1
0.9
37
0.9
33
NS
6
0
TH2
4.3
40
4.8
36
NS
12
6
0
GFP
CD44
Bcl6: TG WT
GFP+MP
B
Starting
TH0
TH1
TH2
MFI of GFP
300
200
100
0
**
*
NS
**
†
Bcl6: TG WT TG WT TG WT TG WT
GFP+MP

Fig S2

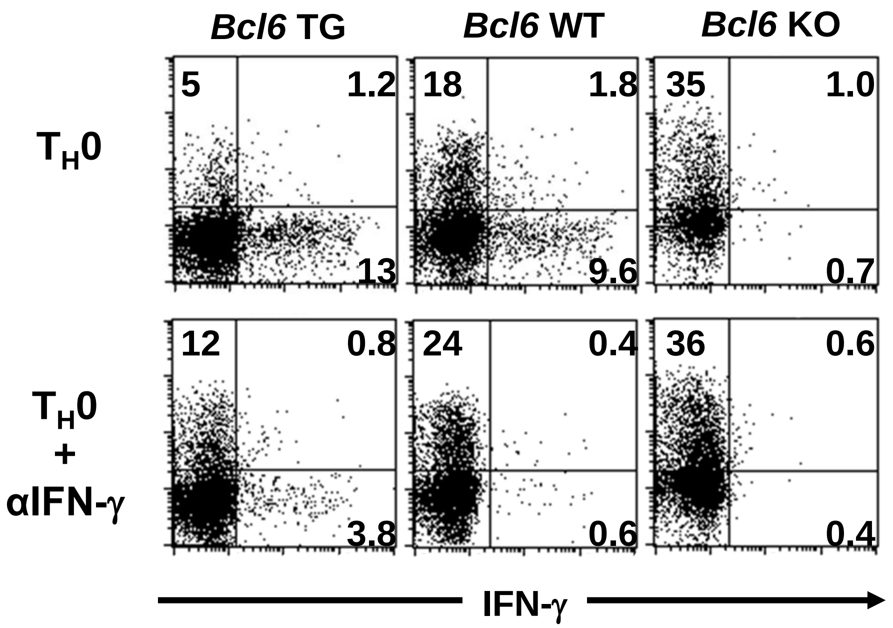

Bcl6 TG
Bcl6 WT
Bcl6 KO
TH0
TH0 + αIFN-γ
5
1.2
13
18
1.8
9.6
35
1.0
0.7
12
0.8
3.8
24
0.4
0.6
36
0.6
0.4
IL-4
IFN-γ

Fig S3

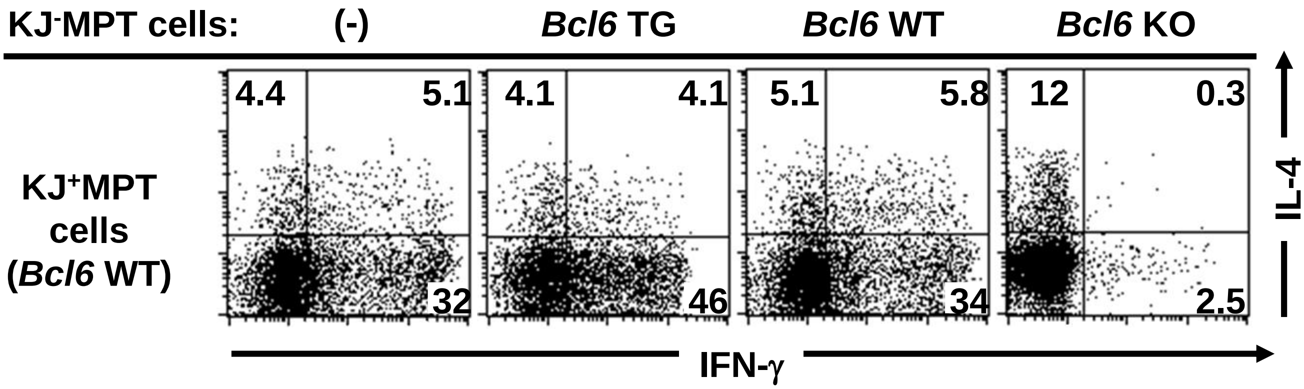

KJ-MPT cells:
(-)
Bcl6 TG
Bcl6 WT
Bcl6 KO
KJ+MPT cells (Bcl6 WT)
4.4
5.1
32
4.1
4.1
46
5.1
5.8
34
12
0.3
2.5
IL-4
IFN-γ

Fig. S4

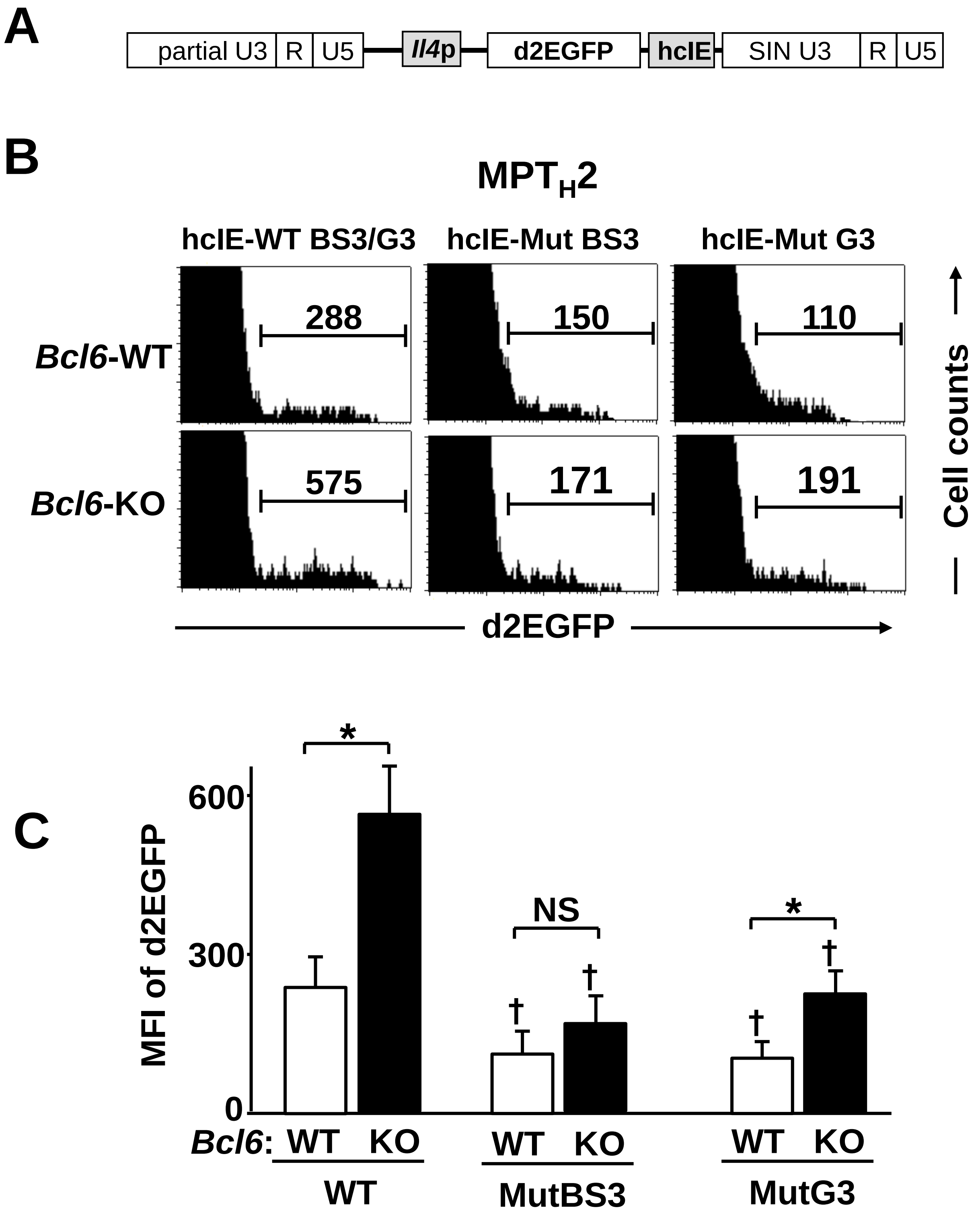
A
partial U3
R
U5
Il4p
d2EGFP
hcIE
SIN U3
R
U5
B
MPT$_H$2
hcIE-WT BS3/G3
hcIE-Mut BS3
hcIE-Mut G3
Bcl6-WT
Bcl6-KO
288
150
110
575
171
191
Cell counts
d2EGFP
C
MFI of d2EGFP
600
300
0
*
NS
*
†
Bcl6: WT KO
WT KO
WT KO
WT
MutBS3
MutG3

Fig. S5

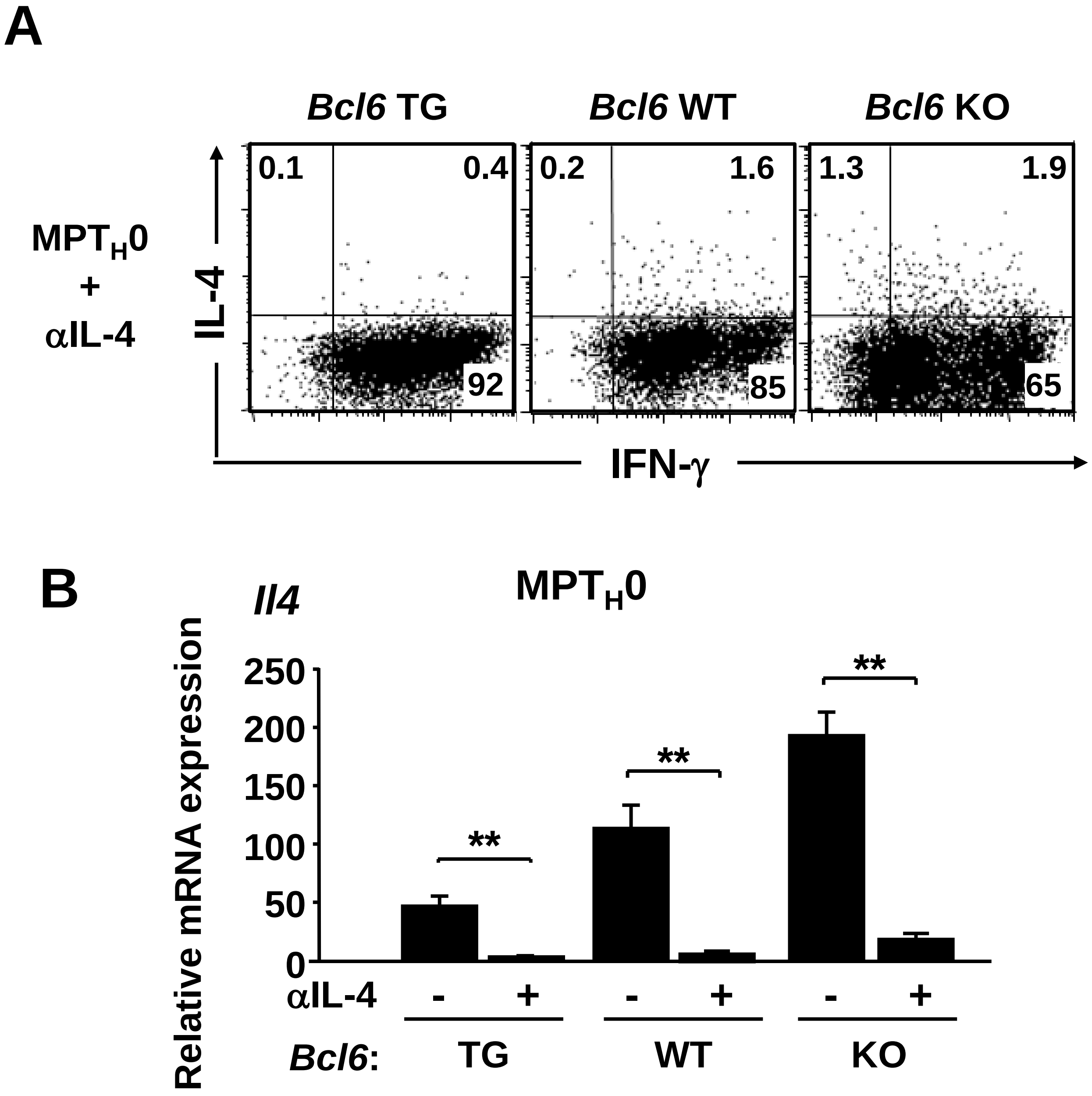
A
Bcl6 TG
Bcl6 WT
Bcl6 KO
MPTH0
+
αIL-4
IL-4
IFN-γ
0.1
0.4
92
0.2
1.6
85
1.3
1.9
65
B
Il4
MPTH0
Relative mRNA expression
250
200
150
100
50
0
**
**
**
αIL-4
-
+
-
+
-
+
Bcl6:
TG
WT
KO

Fig. S6

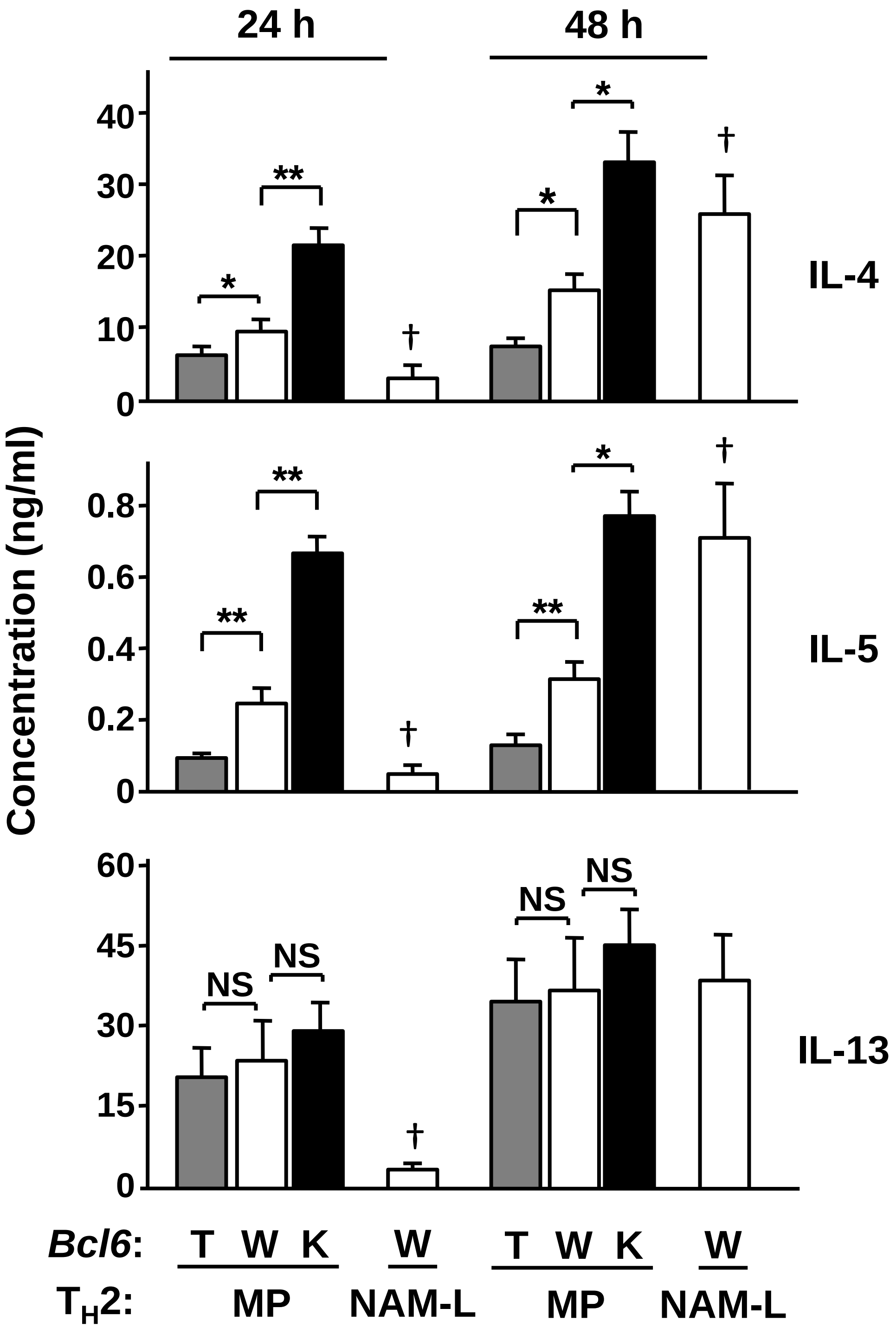

24 h
48 h
40
30
20
10
0
**
*
*
*
†
†
IL-4
Concentration (ng/ml)
0.8
0.6
0.4
0.2
0
**
**
**
*
†
†
IL-5
60
45
30
15
0
NS
NS
NS
NS
†
IL-13
Bcl6: T W K W T W K W
TH2: MP NAM-L MP NAM-L

Original data of western blotting for Bcl6 protein

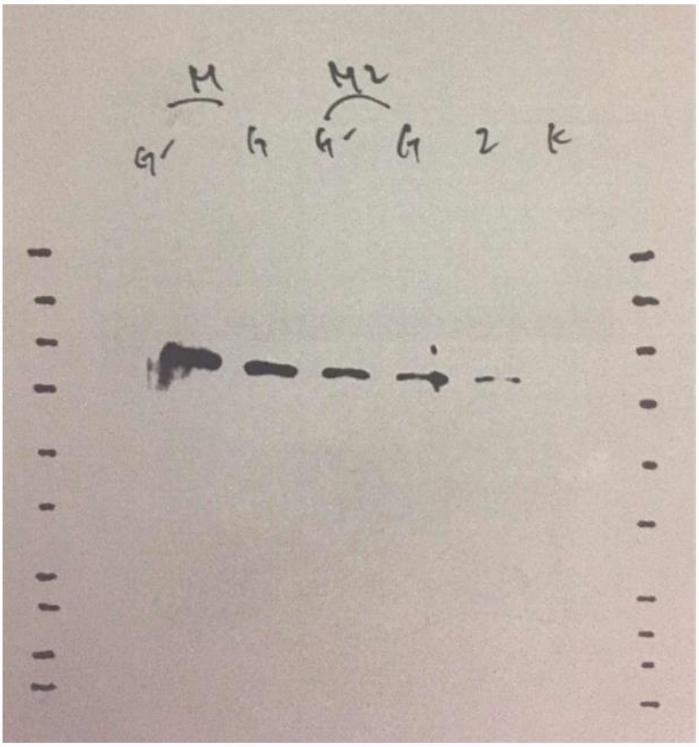

(KDa)
250
150
100
75
50
